# To Control False Positives in Gene-Gene Interaction Analysis: Two Novel Conditional Entropy-Based Approaches

**DOI:** 10.1371/journal.pone.0081984

**Published:** 2013-12-10

**Authors:** Xiaoyu Zuo, Shaoqi Rao, An Fan, Meihua Lin, Haoli Li, Xiaolei Zhao, Jiheng Qin

**Affiliations:** 1 Department of Medical Statistics and Epidemiology, Sun Yat-Sen University, Guangzhou, China; 2 Institute of Medical Systems Biology and Department of Medical Statistics and Epidemiology, Guangdong Medical College, Dongguan, China; National Institute of Environmental Health Sciences, United States of America

## Abstract

Genome-wide analysis of gene-gene interactions has been recognized as a powerful avenue to identify the missing genetic components that can not be detected by using current single-point association analysis. Recently, several model-free methods (e.g. the commonly used information based metrics and several logistic regression-based metrics) were developed for detecting non-linear dependence between genetic loci, but they are potentially at the risk of inflated false positive error, in particular when the main effects at one or both loci are salient. In this study, we proposed two conditional entropy-based metrics to challenge this limitation. Extensive simulations demonstrated that the two proposed metrics, provided the disease is rare, could maintain consistently correct false positive rate. In the scenarios for a common disease, our proposed metrics achieved better or comparable control of false positive error, compared to four previously proposed model-free metrics. In terms of power, our methods outperformed several competing metrics in a range of common disease models. Furthermore, in real data analyses, both metrics succeeded in detecting interactions and were competitive with the originally reported results or the logistic regression approaches. In conclusion, the proposed conditional entropy-based metrics are promising as alternatives to current model-based approaches for detecting genuine epistatic effects.

## Introduction

Over the past years, genome-wide association analysis has greatly facilitated the identification of common genetic factors that were relevant to complex diseases. Based on the common disease common variants hypothesis [1], [2], [3], the single-point association approaches, as a conventional technique, were prevailing in detecting predominant risk variants. Although abundant susceptible common variants have been identified, their effectiveness in interpreting and dissecting complex genetic architectures predisposing to complex diseases remains limited. Only a small proportion of disease heritability can be accounted for, with a great large number of trigger variants that interplay with the main genetic factors being untouched [4], [5]. As a growing conception in recent years, most complex diseases are primarily resulted from the dynamic processes of the biological networks (e.g. pathways) involving multiple gene-gene and gene-environment interactions [6]. Genome-wide analysis of gene-gene interactions has been deemed as an inevitable avenue to identify the more involved genetic components contributing to complex diseases that are undetectable in current single-point association analysis frameworks [7], [8], [9], [10].

Numerous methods or algorithms for detecting gene-gene interactions have been developed. In general, they can be categorized into two classes: model-based and model-free, based on whether a explicit interaction model is assumed. The former is to explicitly model and estimate, e.g. the odds ratio or relative risk, to measure the interaction effects in a dichotomous phenotype (e.g. affected or unaffected). This class of methods is usually based on the generalized linear model framework, and is superior when the true interaction pattern is prior known. However, substantial bias and power loss might occur if the model is incorrectly assumed, which is often the case in practice where the true model is unknown. In this sense, a model-free method has its own merits (*i.e.* robust to model misspecifications) being used in a genome-wide interaction analysis. Random forest [11], ensemble decision tree [12], multi-dimensionality reduction [13] and entropy-based methods [14], [15], [16], [17] are widely used model-free techniques for detecting the epistatic effects or more strictly the joint effects between genetic loci.

The entropy-based methods have received increased attention in recent years. Several entropy-based statistics or metrics have been developed for detecting non-linear dependence [18], [19] in contingency table, or synergy [20] between genetic loci in association studies, and have shown their power in detecting interaction [14], [15], [16], [17]. Mutual information (MI), a metric measuring the dependence between two attributes [21], has been widely applied as a metric or integrated in the ensemble approaches for an interaction analysis [17], [22], [23], [24], for its merits of capturing non-linear dependence and the model-free nature. One typical application is the mutual information based on the joint genotype distribution (named as *GenoMI* in this study), which measures the association between a SNP pair (e.g. loci *G* and *H*) and the disease status (*D*). By defining *S* as the joint genotypes of *G* and *H*, which have 9 possible values, *GenoMI* can be formulated as: 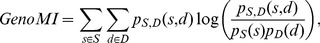
(1)where 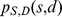, 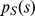 and 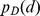 are the joint probability mass functions of *S* and *D*, marginal probability mass functions of *S* and *D*, respectively. If cases and controls have the same joint genotype distribution, *GenoMI* equals to zero. With large sample size, *GenoMI* approximately follows 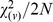 [25], where *N* is the total sample size and the degree of freedom, *v*, equals to 8. *GenoMI* is plausible to detect interaction because interaction can generate discrepancies in joint genotype distribution between cases and controls. Indeed, *GenoMI* has shown its power for constructing genetic association network for rheumatoid arthritis [26].

However, many entropy-based metrics have potentially suffered from a risk of inflated false positive error (type 1 error) in testing the interaction hypothesis, induced by the main effects of loci. In an interaction analysis, the false positive error is an undesirable error and requires great efforts to control. Nevertheless, this issue has received limited attention. Very recently, it was in detail discussed by Ueki and Cordell [27], who systematically investigated the inflated type 1 error associated with several existing methods, for example the logistic regression-related metrics. Theoretically, the joint effect of two loci predisposing to disease could be decomposed into the main effects for the individual loci and the interaction effects derived from some specific genotypic or allelic combinations between the loci. Based on a generalized linear model (e.g. logistic regression model), the main effects and the interaction effect for two loci *G*_1_ and *G*_2_ can be expressed as: 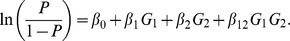



A reasonable way to detect interactions is to partition the joint effect of the two loci and directly test whether *β*_12_ equals to 0, which, however, is very complicated in model-free methods. Some widely used model-free methods are not able to distinguish the interaction effect from the joint effect. For example, the rationale for *GenoMI* to detect interaction is the presence of the interaction-induced variation in the joint genotypic distribution between cases and controls. However, such genotypic fluctuation can also be generated by the main effect, even if the interaction does not exist. Thus, there is a potential risk of inflated false positive error in *GenoMI*, as well as other entropy-based statistics, in the presence of main effect. This problem and its solution, unfortunately, have not been well addressed.

Inspired by the work of Ueki and Cordell [27], who investigated this issue associated with the model-based approaches, we attempted to solve this problem in the model-free settings. In this study, we first showed the problem of inflated false positive in using *GenoMI* metric to detect interaction via extensive computer simulations. Then, to remedy this problem, we proposed and derived two conditional mutual information (CMI) based metrics (*i.e.* based on genotypic information metric, named as *GenoCMI*, and based on gametic information metric, named as *GameteCMI*, respectively) to enhance the capability of the information-based metrics to control type 1 error induced by the main effect. Finally, we systematically evaluated the two proposed metrics for detecting genuine interactions (epistasis), in terms of type 1 error control and statistical power, by using large-scale simulations and applications to two real data examples.

## Materials and Methods

### CMI metric based on genotype

CMI is a fundamental concept in information theory, defined as the reduction of the uncertainty of variable *X* due to the knowledge of variable *Y* when variable *Z* is given. In statistical perspective, the null hypothesis under CMI is conditional independence, assuming that *X* and *Y* are mutually independent across each stratum of *Z*.

Denote allele *A* and *B* as the risk alleles of the unlinked SNP loci *G* and *H*, respectively. Let *i* and *j* (*i*, *j* = 0, 1 or 2) represent the genotypes of loci *G* and *H*, respectively, based on the count of risk alleles. Let *D* denote the disease status, where *D* = 1 (*D* = 0) indicates affected (unaffected). CMI based on genotypes (*GenoCMI*) between two SNP loci can be defined as 
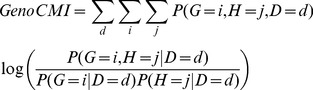
(2)


Suppose that the case-control study has *P_A_* cases and 1-*P_A_* controls. Let *P_ij_*, *P_i_*_._, and *P*_.*j*_ be the joint genotype frequency *P*(*G* = *i*, *H* = *j*), the marginal frequencies *P*(*G* = *i*) and *P*(*H* = *j*), respectively, in the general population. Let *K* be the disease prevalence. Then, after some algebra (for the detailed derivation, see Text S1), equation (2) can be expressed as: 
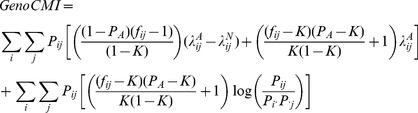
(3)where 
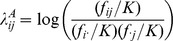
, and 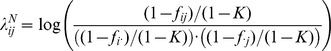. 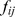, 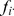 and 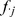 are the penetrance values of joint genotype *i* and *j*, genotype *i* and genotype *j*, respectively. 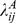 measures the logarithmic departure of the relative risk of joint genotype (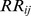) from the product of the relative risks of their corresponding marginal genotypes (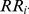 and 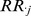), which implies that no interaction effect between *i* and *j* exits if and only if 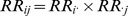 [17]. Hence, it corresponds to the definition of multiplicative interaction defined in terms of relative risk. The latter term in equation (3) can reduce to zero if loci *G* and *H* are independent in general population, for example, they are in linkage equilibrium (or unlinked). Note that
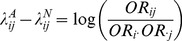
(4)is another commonly applied metric for the interaction defined by odds ratio (OR), where 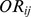, 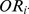 and 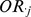 are the odds ratios of the joint genotype i and j and their corresponding marginal genotypes, respectively. Under the rare disease assumption, the equation (3) can be further reduced to (for the detailed derivation, see Text S1):



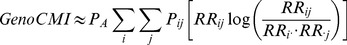
(5)The equation (5) indicates that the *GenoCMI* metric measures the quantities of interactions between genotypes of loci *G* and *H* in terms of relative risk. If the interaction between genotypes *i* and *j* is absent, the relative risk of the joint genotype *ij* is expected to equal the multiplication of their marginal relative risks such that *GenoCMI* equals to zero. On the other hand, any departure of *GenoCMI* from zero indicates the presence of interaction between two loci. Under the null hypothesis, the *GenoCMI* metric approximately follows 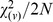 distribution where the degree of freedom (*v*) is 8 and *N* is number of individuals (see Text S2) [18].

### CMI metric based on gametic disequilibrium

Several previous studies have successfully utilized gametic disequilibrium as a measure to detect interaction [28], [29], [30], [31], [32]. Gametic disequilibrium in turn has a direct impact on the entropy of gamete [16]. Inspired by these findings, we developed a similar gamete-based metric (*GameteCMI*) to the abovementioned *GenoCMI* to measure conditional allelic dependence between two unlinked loci. Consider two unlinked loci *G* and *H* with alleles *A* and *B* being the risk alleles. Let *h_kl_* be a gamete of loci *G* and *H*, where *k* and *l* (*k*, *l* = 0 and 1) indicates the carrier states of the risk alleles *A* and *B* in the gamete, respectively. The four possible gamete for the diallelic loci *G* and *H*, *h*_00_, *h*_01_, *h*_10_ and *h*_11_, represent for gamete *ab*, *aB*, *Ab* and *AB*, respectively. Analogous to equation (2), the *GameteCMI* metric can be defined as: 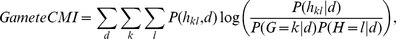
(6)where *P*(*G* = *k*|*d*) and *P*(*H* = *l*|*d*) are the frequencies of alleles *k* and *l* under disease status *d*, respectively. Under the rare disease assumption, the equation (6) could be reduced to (see Text S1 for details):
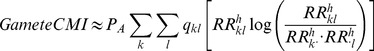
(7)where 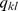, 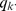 and 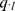 are the frequencies of gamete hkl, and alleles k and l in general population, respectively. 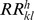, 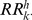 and 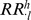 are the relative risk of gamete hkl, and alleles k and l, respectively. In contrast to GenoCMI, GameteCMI measures allelic interaction between two loci. If there is no allelic interaction, the joint risk of k-l allele pair (e.g. gamete k-l) is of no logarithmic departure from the multiplication of their marginal risks such that GameteCMI equals to zero. Otherwise, the amount of this departure indicates the strength of interaction between the alleles of the two loci. Under the null hypothesis of no allelic interaction, GameteCMI asymptotically follows 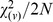, where the degree of freedom (v) is 2 and N is number of individuals (see Text S2 for the derivation).

### Simulation methods

To evaluate the two proposed metrics, we performed extensive simulations in varieties of parameter settings. The publicly available software, genomeSIMLA [33], was used to generate multi-loci genotypic data for case-control design. The simulations first generated two chromosomes, one containing 2 LD blocks and the other containing 1 block. In each LD block, there were 10 SNP loci, and the recombination rates between blocks and within blocks were randomly chosen between 0.00006 and 0.0004, and between 0.00000001 and 0.0000001, respectively. After thousands of generations simulated, we randomly chose the 2050th generation with 1,000,000 individuals as the general population. Then, we assigned two SNP loci (*G* and *H*) located on different chromosomes to be the disease loci for a dichotomous disease phenotype. The risk allele frequencies at loci *G* and *H* in the general population were 0.30 and 0.42, respectively. Table 1 summarized the simulation schemas performed in this study. For each schema, varieties of parameters were simulated and 1000 replicates were generated for each parameter setting.

**Table 1:** Description of simulation schemas.

Schema^a^	*OR_G_* b	*OR_H_* b	*OR_GH_* b	*K* c	Description
1	–	–	–	0.02	Global null hypothesis of no main effect and interaction effect
2	√	–	–	0.02	Only locus *G* has a main effect (*OR_G_* = 2.0 and 3.0, respectively). Assume a common disease model.
3	√	√	–	0.02	Both loci have main effects (*OR_G_* = *OR_H_* = 2.0 and 3.0, respectively). Assume a common disease model.
4	√	–	–	0.0001	Similar to 2, but assuming a rare disease model.
5	√	√	–	0.0001	Similar to 3, but assuming a rare disease model.
6	√	–	–	0.02	Similar to 2, but assuming a 1∶2 case/control ratio (*P_A_* = 1/3).
7	√	√	–	0.02	Similar to 3, but assuming a 1∶2 case/control ratio (*P_A_* = 1/3).
8^d^	–	–	√	0.02	Loci *G* and *H* have an interaction effect, but no main effect at both loci.
9^d^	√	–	√	0.02	Loci *G* and *H* have an interaction effect, with main effect at locus *G* (*OR_G_* = 2.0).

a In each schema, three two-locus interaction models (additive × additive, dominant × dominant and recessive × recessive) were evaluated.

b *OR_G_*, *OR_H_*, and *OR_GH_* denote the main effect for locus *G*, main effect for locus *H*, and their interaction effect, respectively. “√” indicates that the effect is present. “–” indicates that the effect is absent.

c Disease prevalence (baseline penetrance).

d For Schemas 8 and 9, the interaction effect *OR_GH_* was increased from 1.0 to a value at which the power of the optimal metric achieved 100% at significance level 0.01.

Schemas 1–7 were used to assess the capability of the two proposed metrics to control type 1 error under varieties of parameter settings. Schema 1 assumed lack of both main effects and interaction effects at both loci (global null hypothesis). Schema 2 assumed that only one locus had main effect, and Schema 3 assumed that both loci had main effects. Both schemas were assumed under a common disease assumption (prevalence *K* = 0.02) and 1∶1 case/control ratio (1000 cases and 1000 controls). To assess the influence of disease prevalence, a rare disease assumption with prevalence *K* = 0.0001 (in Schemas 4 and 5) was also considered. Moreover, to assess the influence of case/control ratio, a 1∶2 design with 1000 cases and 2000 controls was carried out (in Schemas 6 and 7). Finally, for power evaluation, two schemas were designed. In Schema 8, neither of the loci had main effect, and in Schema 9, one locus had a main effect.

### Comparisons with alternative methods

We compared our proposed metrics with several previously developed statistics. Original Wu et al. statistic [29], the adjusted Wu statistic [27], and the joint effect statistic [27] were derived from the generalized linear model framework. *GenoMI*, the unconditional information metric, is a widely used metric integrated with several data mining approaches. In addition, we used two logistic regression models as the benchmark references, which theoretically is able to achieve the best performance under the correct genetic model. It should keep in mind that such an analysis is unachievable in practice, as we do not know the true model. The first model coded genotypes at both loci and their interaction patterns (*i.e.* additive × additive, dominant × dominant, or recessive × recessive model) as the truly simulated, then used a 1 df Wald test (named logit_1df in this study) to test the only interaction term. The second model coded genotypes at each locus as two independent factor levels, and as a result, this model had four independent interaction terms (*i.e.* a saturated model). Therefore, the Wald test (named logit_4df) had 4 degrees of freedom. In essence, the second model can be considered “model-free”. Several methods (*GameteCMI*, original Wu et al. and adjusted Wu statistics) require estimation of gamete frequencies, which was carried out by using the E-M algorithm implemented in R library “haplo.stats” (version 1.5.6). The nominal significance of 0.01 was used as the criterion as no multiple tests in each round of simulation were involved. The empirical type 1 error rate or the statistical power was calculated as the percentage of significant results (*P*<0.01) among the 1000 replicates.

## Results

### The Null Distributions of the CMI-based Metrics

**Figure 1:**
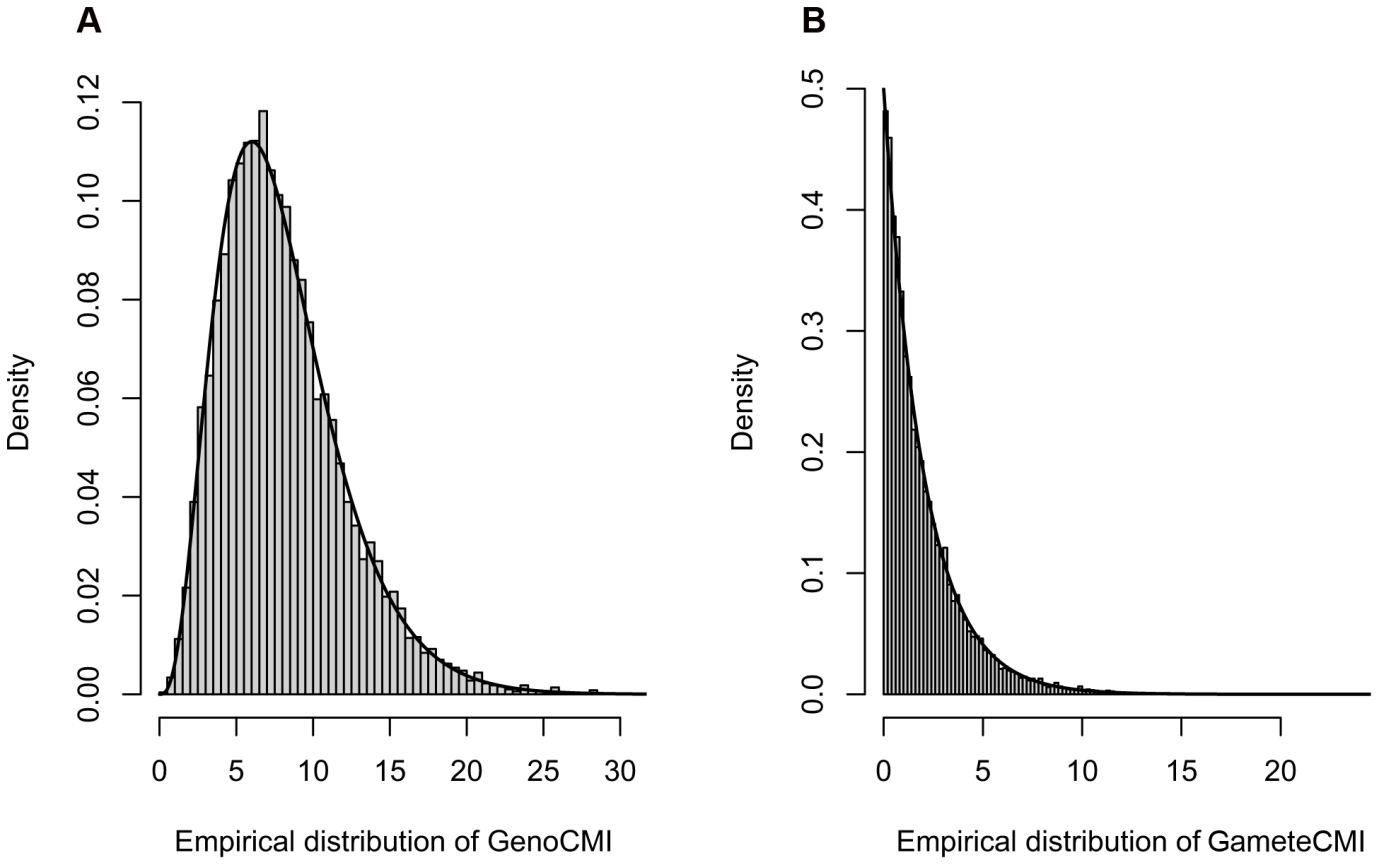
Null distribution of the *GenoCMI* and *GameteCMI* metrics. **A**. The empirically null distribution of *GenoCMI*, compared to its theoretical distribution *χ* ^2^_(8)_. **B**. The empirically null distribution of *GameteCMI*, compared to its theoretical distribution *χ* ^2^_(2)_.

To empirically determine the asymptotic distributions of the CMI-based metrics (*GenoCMI* and *GameteCMI*), we first simulated a general population of 1,000,000 individuals with two independent diallelic loci. Then, we randomly sampled 1,000 individuals from the general population and randomly assigned their disease status (independent of genotypes) according to the specified population prevalence. We repeated 10,000 times to obtain the empirical distributions of *GenoCMI* and *GameteCMI* and compared with their corresponding theoretical distributions, the central *χ* ^2^_(8)_ and *χ* ^2^_(2)_, respectively (Figure 1). The Kolmogorov-Smirnov test indicated good goodness-of-fitness of the empirical distributions of *GenoCMI* (*P* = 0.961) and *GameteCMI* (*P* = 0.848) with their theoretical ones, respectively.

### Type 1 Error Without Any Main Effect

**Figure 2:**
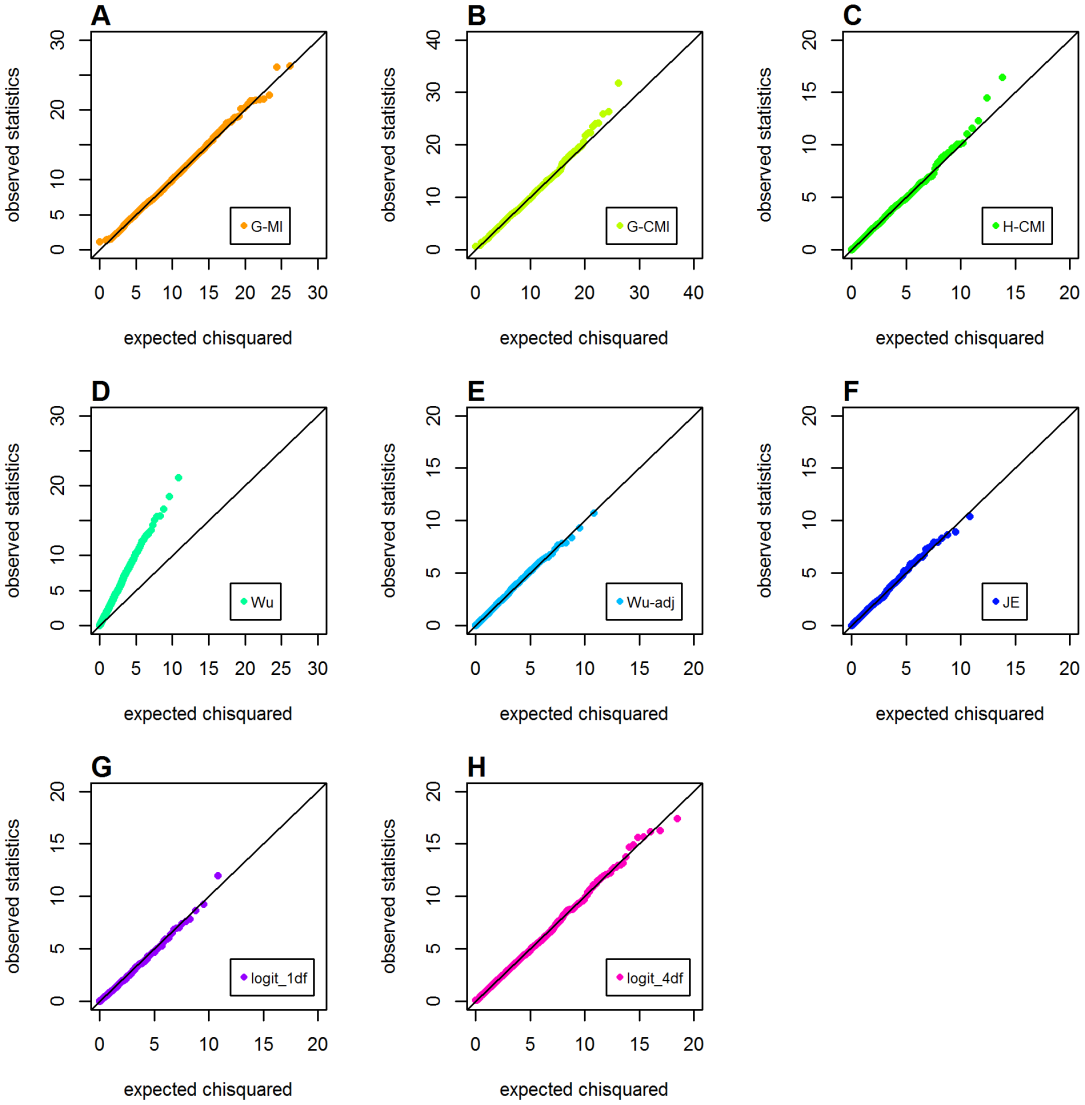
Chi-squared Q-Q plots for the global null hypothesis (Schema 1). Top panels: **A**. *GenoMI*; **B**. *GenoCMI*; **C**. *GameteCMI*. Middle panels: **D**. original Wu et al statistic; **E**. adjusted Wu statistic; **F**. joint effect statistic. Bottom panel: **G**. logistic regression model with 1 df test; **H**. logistic regression model with 4 df test.

Figure 2 shows Q-Q plots for the observed distributions of different metrics calculated in Schema 1 (with the global null hypothesis of no main effects and interaction effects). All metrics performed well in this schema, except for the original Wu et al. statistic that showed obvious inflation in type 1 error, which was consistent with Ueki and Cordell's findings [27].

### Type 1 Error with One Locus Having a Main Effect

**Figure 3:**
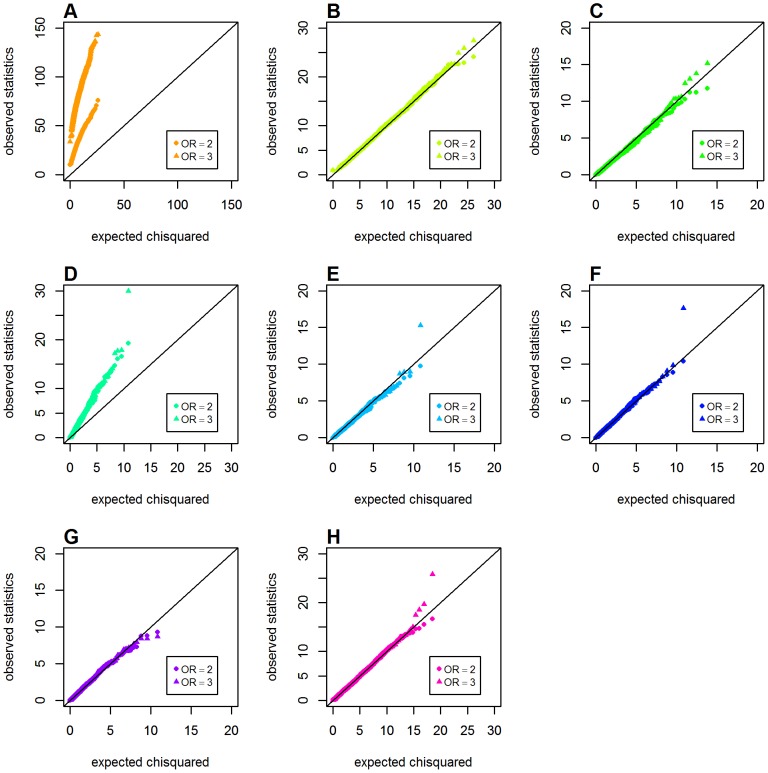
Chi-squared Q-Q plots for the recessive model with main effect at one locus (Schema 2). Top panels: **A**. *GenoMI*; **B**. *GenoCMI*; **C**. *GameteCMI*. Middle panels: **D**. original Wu et al statistic; **E**. adjusted Wu statistic; **F**. joint effect statistic. Bottom panel: **G**. logistic regression model with 1 df test; **H**. logistic regression model with 4 df test.

We first evaluated the issue of type 1 error for the two proposed CMI-based metrics in Schema 2, in which only one locus (*G*) had a main effect. The *GenoCMI* and *GameteCMI* showed good agreement between the observed and expected values under two different main effect settings (*OR_G_* = 2.0 and 3.0, respectively). However, an obvious departure was identified for the *GenoMI* metric when one locus (*G*) had a main effect, supporting our perspective that *GenoMI* is unable to distinguish the genuine interaction effect from the joint effect well. All three logistic-based statistics also showed satisfactory performance in these parameter settings except for the original Wu et al. statistic that showed a marked deviation. Except for *GenoMI*, genetic models appeared having only subtle influence on various metrics to control type 1 error, with *GameteCMI* and adjusted Wu statistic showing slight inflation in the dominant model when *OR_G_* = 3.0. (see Figure 3 for the recessive model, Figure S1 for the dominant model and Figure S2 for the additive model).

The estimated type 1 error rates (at *α* = 0.01) for testing interaction in a common disease model (*K* = 0.02) are given in Table 2. It is evident that *GenoMI* could not maintain correct type 1 error when one locus had a main effect, indicating that it is very sensitive to any departure from the global null hypothesis of no main effect at both loci and no interaction effect. It is also interesting to note that the original Wu et al. statistic had a fairly consistently inflated type 1 error over a range of main effects and genetic models. All the remaining six metrics or statistics, including our proposed two conditional information based metrics, adjusted Wu statistic, the joint effect statistic, the logistic regression model with 1 df Wald test and the logistic regression model with 4 df Wald test, had well maintained correct type 1 error rates over three common genetic models and different sizes of main effect. In short, these simulations demonstrated that the two proposed CMI-based metrics could tolerate the influence on type 1 error rate from the large main effects (e.g. *OR* = 3) at one locus.

**Table 2:** False positive rates (type 1 error rates) for testing interaction in common disease with main effect at one locus (Schema 2).

	Additive			Dominant			Recessive		
	1.0	2.0	3.0	1.0	2.0	3.0	1.0	2.0	3.0
*GenoMI*	0.009	1.000	1.000	0.013	1.000	1.000	0.006	0.913	1.000
*GenoCMI*	0.009	0.008	0.010	0.011	0.014	0.009	0.007	0.011	0.011
*GameteCMI*	0.005	0.003	0.011	0.012	0.015	0.018	0.015	0.007	0.010
original Wu statistic	0.063	0.086	0.069	0.074	0.094	0.088	0.079	0.064	0.061
adjusted Wu statistic	0.004	0.011	0.006	0.009	0.019	0.018	0.012	0.006	0.007
joint effect statistic	0.004	0.009	0.006	0.010	0.016	0.010	0.016	0.010	0.008
logit_1df^a^	0.005	0.014	0.007	0.009	0.016	0.013	0.011	0.009	0.010
logit_4df^b^	0.009	0.006	0.010	0.008	0.014	0.014	0.009	0.009	0.009

a logistic regression model with 1 df test for the correct genetic model.

b logistic regression model with 4 df test by coding genotypes as factors.

The disease prevalence is assumed 0.02. The significance level is set as 0.01.

### Type 1 Error with Both Loci Having Main Effects

**Figure 4:**
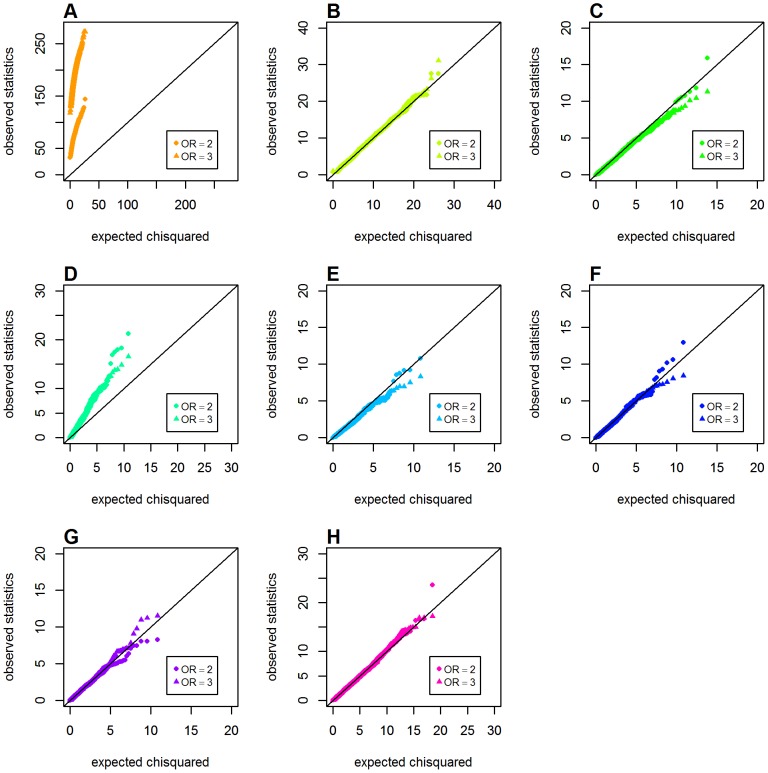
Chi-squared Q-Q plots for the recessive-recessive model with main effect at both locus (Schema 3). Top panels: **A**. *GenoMI*; **B**. *GenoCMI*; **C**. *GameteCMI*. Middle panels: **D**. original Wu et al statistic; **E**. adjusted Wu statistic; **F**. joint effect statistic. Bottom panel: **G**. logistic regression model with 1 df test; **H**. logistic regression model with 4 df test.

**Figure 5:**
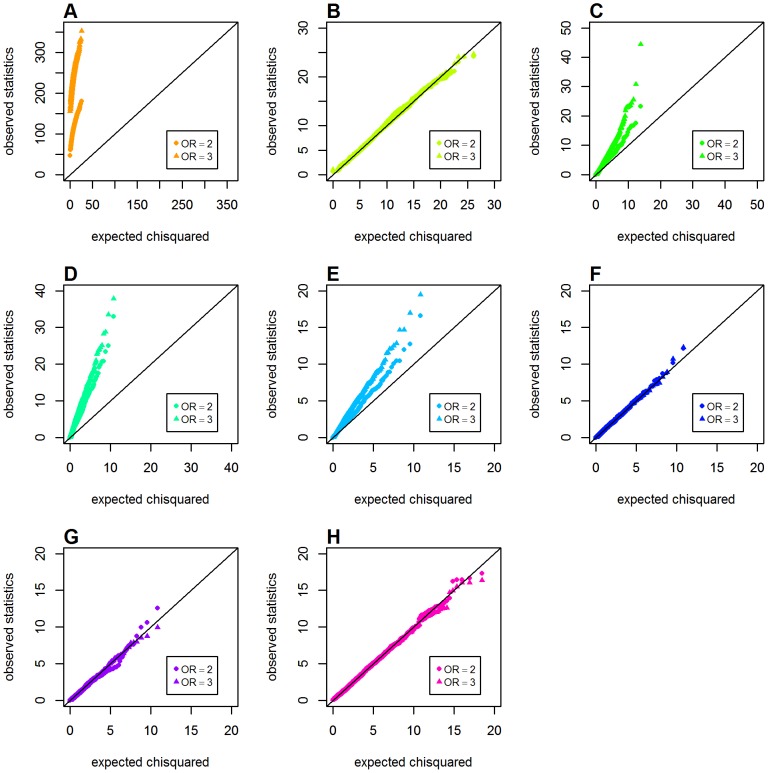
Chi-squared Q-Q plots for the dominant-donimant model with main effect at both locus (Schema 3). Top panels: **A**. *GenoMI*; **B**. *GenoCMI*; **C**. *GameteCMI*. Middle panels: **D**. original Wu et al statistic; **E**. adjusted Wu statistic; **F**. joint effect statistic. Bottom panel: **G**. logistic regression model with 1 df test; **H**. logistic regression model with 4 df test.

**Figure 6:**
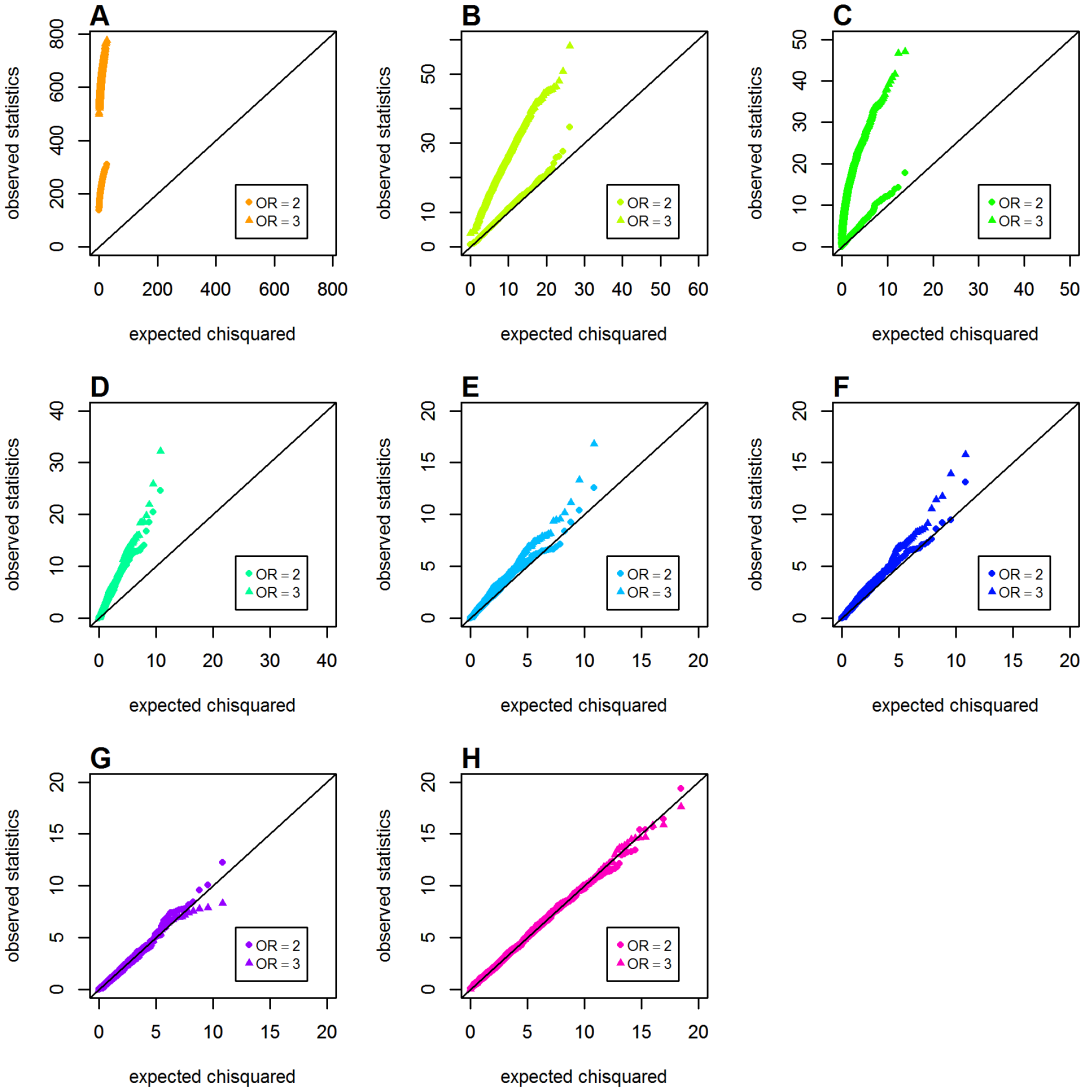
Chi-squared Q-Q plots for the additive-additive model with main effect at both locus (Schema 3). Top panels: **A**. *GenoMI*; **B**. *GenoCMI*; **C**. *GameteCMI*. Middle panels: **D**. original Wu et al statistic; **E**. adjusted Wu statistic; **F**. joint effect statistic. Bottom panel: **G**. logistic regression model with 1 df test; **H**. logistic regression model with 4 df test.

We further investigated the issue of type 1 error in detecting interactions when both loci had main effects under a common disease model (disease prevalence *K* = 0.02). Figure 4 shows the Q-Q plots for Schema 3 under a recessive-recessive two-locus disease model. Again, markedly inflated type 1 error was observed for *GenoMI* and the original Wu et al. statistic. All the remaining metrics or statistics, including the proposed *GenoCMI* and *GameteCMI*, had maintained the correct type 1 error rate. However, in the dominant-dominant interaction model, the gamete-based statistics (*GameteCMI* and the adjusted Wu statistic) appeared not being so effective to maintain the correct type 1 error as well. This inflation was more salient when the sizes of main effect became larger (see Figure 5). In this case *GenoCMI* and the joint effect statistic were comparable to the two logistic regression models. Finally, the inflation of type 1 error was most pronounced under the additive-additive interaction model, in which none of the tested approaches could maintain correct type 1 error rate except for the logistic regression with 1 df test and the logistic regression with 4 df test (see Figure 6).

### The Influence of Disease Prevalence on Type 1 Error

**Figure 7:**
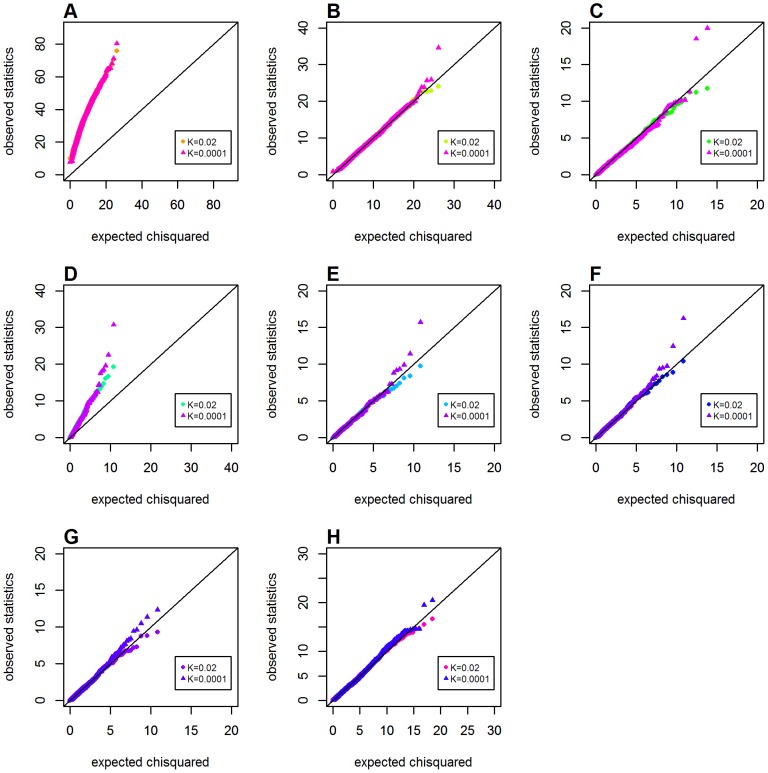
Chi-squared Q-Q plots for the recessive model with main effect at one locus, when disease prevalence varied (Schema 4). Assuming the presence of main effect at one locus (*OR_G_* = 2.0). Top panels: **A**. *GenoMI*; **B**. *GenoCMI*; **C**. *GameteCMI*. Middle panels: **D**. original Wu et al statistic; **E**. adjusted Wu statistic; **F**. joint effect statistic. Bottom panel: **G**. logistic regression model with 1 df test; **H**. logistic regression model with 4 df test.

To assess the influence of disease prevalence (*K*) on the type 1 error rate, we also considered the scenarios of a rare disease (*K* = 0.0001) in Schemas 4 (with one locus having a main effect) and 5 (with both loci having main effects). Figure 7 shows the Q-Q plots with *K* = 0.02, 0.0001 when one locus had a recessive main effect (*OR_G_* = 2.0). The size of *K* appeared to have little effect on the Q-Q plots. A similar pattern also figured in both the dominant model (see Figure S3) and the additive model (see Figure S4), respectively.

**Figure 8:**
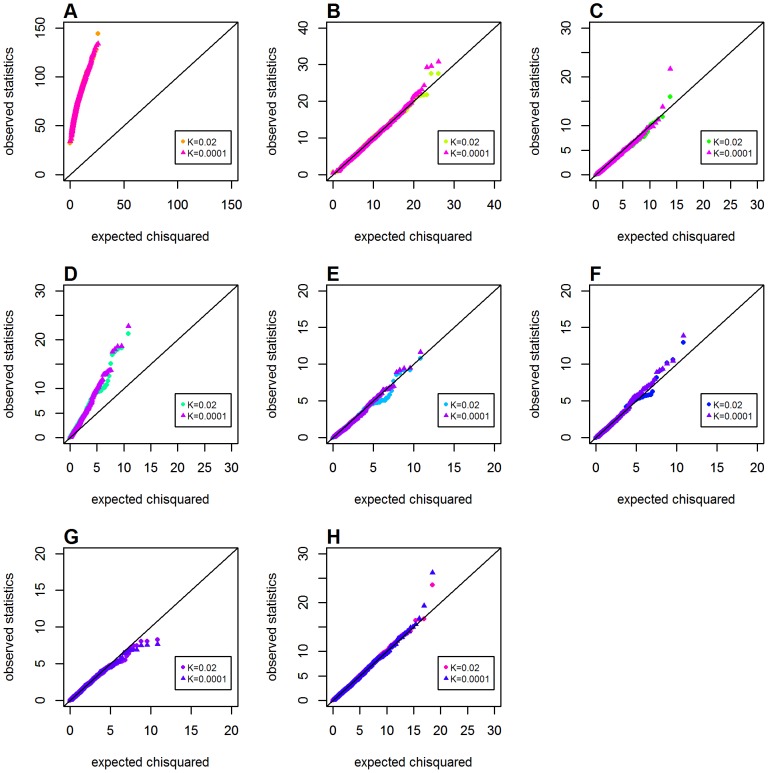
Chi-squared Q-Q plots for the recessive-recessive model with main effect at both loci, when disease prevalence varied (Schema 5). Assuming main effect at both locus (*OR_G_* = *OR_H_* = 2.0). Top panels: **A**. *GenoMI*; **B**. *GenoCMI*; **C**. *GameteCMI*. Middle panels: **D**. original Wu et al statistic; **E**. adjusted Wu statistic; **F**. joint effect statistic. Bottom panel: **G**. logistic regression model with 1 df test; **H**. logistic regression model with 4 df test.

For the situation of both loci having main effects (*OR_G_* = *OR_H_* = 2.0), disease prevalence also had little effect on the Q-Q plots for the recessive-recessive genetic model (see Figure 8) and the ones for the additive-additive model (see Figure S6). However, in dominant-dominant model, *GameteCMI* and the adjust Wu statistic appeared to suffer from consistently inflated type 1 error under different sizes of *K* (see Figure S5), which suggested that these gamete-based approaches were sensitive to the two-locus genetic model, but not to disease prevalence. In short, all metrics or statistics were not sensitive to disease prevalence.

### The Influence of Case/control Ratio on Type 1 Error

**Figure 9:**
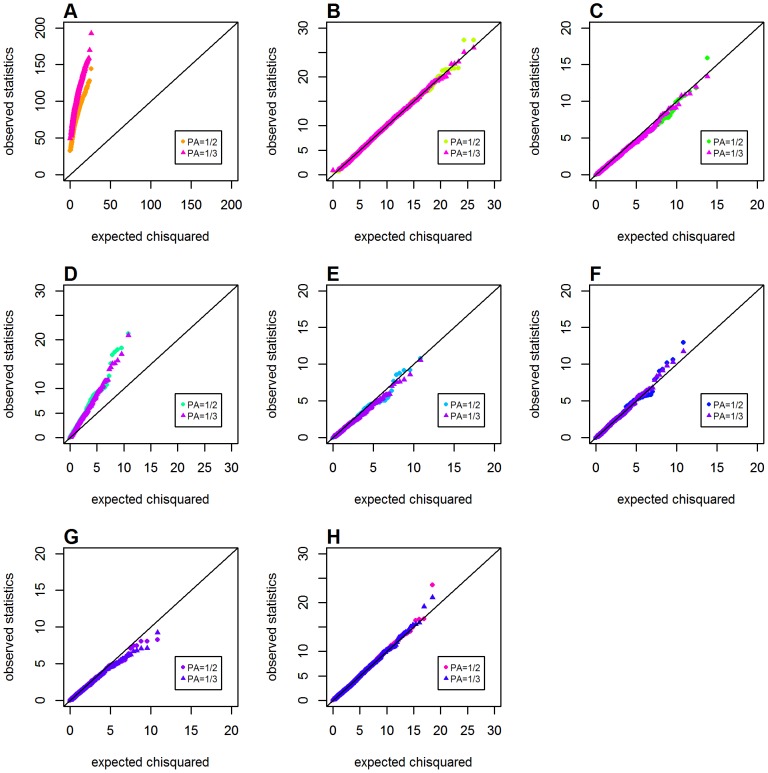
Chi-squared Q-Q plots for the recessive-recessive model with main effect at both loci, when case/control ratios varied (Schema 7). Assuming main effects at both locus (*OR_G_* = *OR_H_* = 2.0) and disease prevalence 0.02. Top panels: **A**. *GenoMI*; **B**. *GenoCMI*; **C**. *GameteCMI*. Middle panels: **D**. original Wu et al statistic; **E**. adjusted Wu statistic; **F**. joint effect statistic. Bottom panel: **G**. logistic regression model with 1 df test; **H**. logistic regression model with 4 df test.

We further evaluated the influence of case/control ratio (*P_A_*) on type 1 error under a common disease model (*K* = 0.02, in Schemas 6 and 7). Little effect of the case/control ratio on Q-Q plots for all seven metrics or statistics was observed in the presence of main effect at one locus (data not shown). When both loci had main effects (*OR_G_* = *OR_H_* = 2.0), only subtle differences in Q-Q plots between different sizes of *P_A_* were observed for different genetic models (see Figure 9 for the recessive-recessive model, Figure S7 for the dominant-dominant model, and Figure S8 for the additive-additive model).

### Power Evaluation

We designed two simulation experiments (Schema 8, without main effect, and Schema 9, with main effect at one locus) for power evaluation. Since the original Wu et al. statistic suffered from the risk of inflated type 1 error even in the scenario of no main effect, we excluded it from power evaluation. Given the fact that *GenoMI* was very sensitive to main effect, we also did not assess its statistical power in the presence of main effect.

**Figure 10:**
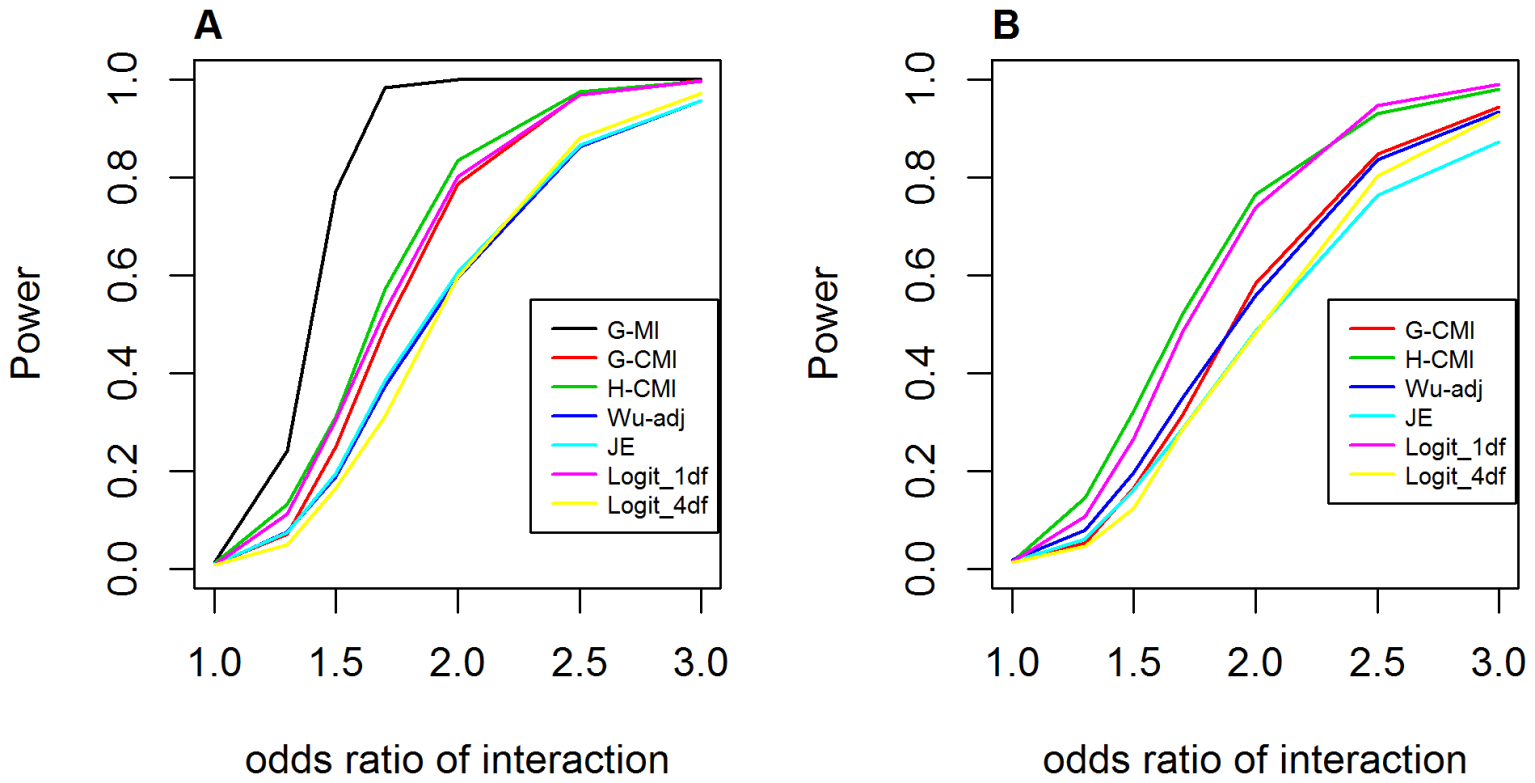
Power curves for testing interaction under the dominant-dominant interaction model. **A**. Assuming no main effect at both loci (*OR_G_* = *OR_H_* = 1.0); **B**. Assuming main effect at one locus (*OR_G_* = 2.0). G-MI: *GenoMI*; G-CMI: *GenoCMI*; H-CMI: *GameteCMI*; Wu-adj: Adjusted Wu statistics; JE: Joint Effects statistics; Logit_1 df: logistic regression model with 1 df test; Logit_4 df: logistic regression model with 4 df test. Disease prevalence was chosen at 0.02.

Figure 10 shows the performance in terms of statistical power for detecting genuine dominant-dominant interaction effects. In absence of main effect at both loci, *GenoMI* appeared to have highest power, followed by *GameteCMI*, the logistic regression with 1 df test and *GenoCMI*. The proposed *GenoCMI* and *GameteCMI* achieved comparable performance to the logistic regression with 1 df test in this scenario, with >80% power at *OR_GH_* = 2.0 or above. The adjusted Wu statistic, the joint effect statistic and the logistic regression with 4 df test appeared to be more conservative in this case. In the presence of main effect at one locus, *GameteCMI* and the logistic regression model with 1 df test achieved the best performance. *GenoCMI* had comparable or relatively higher statistical power compared to the adjusted Wu statistic, the joint effect statistic and the logistic regression model with 4 df test.

**Figure 11:**
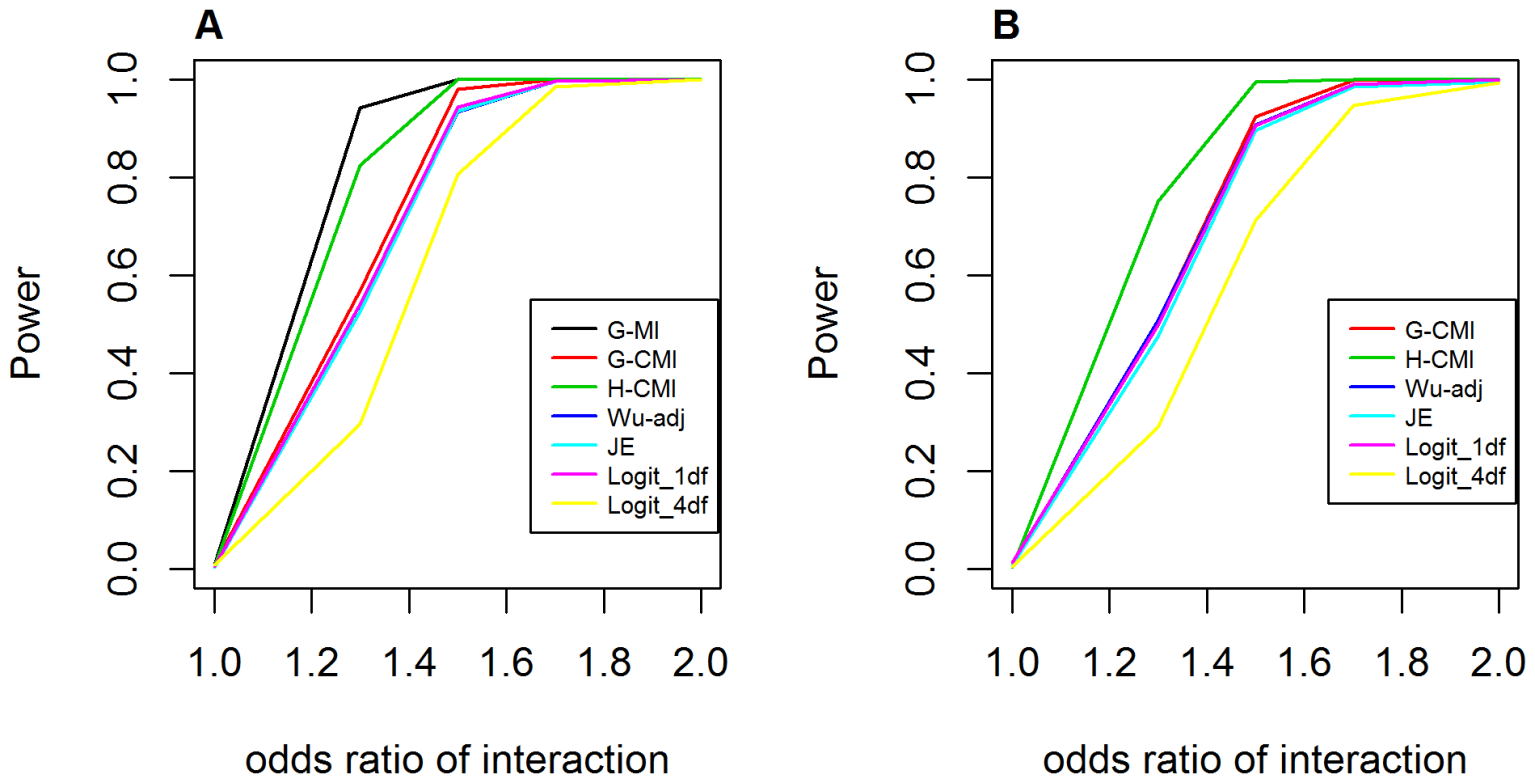
Power curves for testing interaction under the additive-additive interaction model. **A**. Assuming no main effect at both loci (*OR_G_* = *OR_H_* = 1.0); **B**. Assuming main effect at one locus (*OR_G_* = 2.0). G-MI: *GenoMI*; G-CMI: *GenoCMI*; H-CMI: *GameteCMI*; Wu-adj: Adjusted Wu statistics; JE: Joint Effects statistics; Logit_1 df: logistic regression model with 1 df test; Logit_4 df: logistic regression model with 4 df test. Disease prevalence was chosen at 0.02.

Figure 11 shows the power curves for detecting the additive-additive interaction effect. In the case of no main effect at either locus, *GenoMI* outperformed other approaches as well, but was closely followed by *GameteCMI*. *GenoCMI* appeared to have comparable performance to the adjusted Wu statistic, the joint effect statistic and the logistic regression model with 1 df test. The logistic regression model with 4 df test, which did not assume any genetic models, was the most conservative in this case. All metrics or statistics, except for the logistic regression model with a 4 df test for interaction, achieved >90% power at *OR_GH_* = 1.5 or above. The same pattern was also identified in the presence of main effect at one locus.

**Figure 12:**
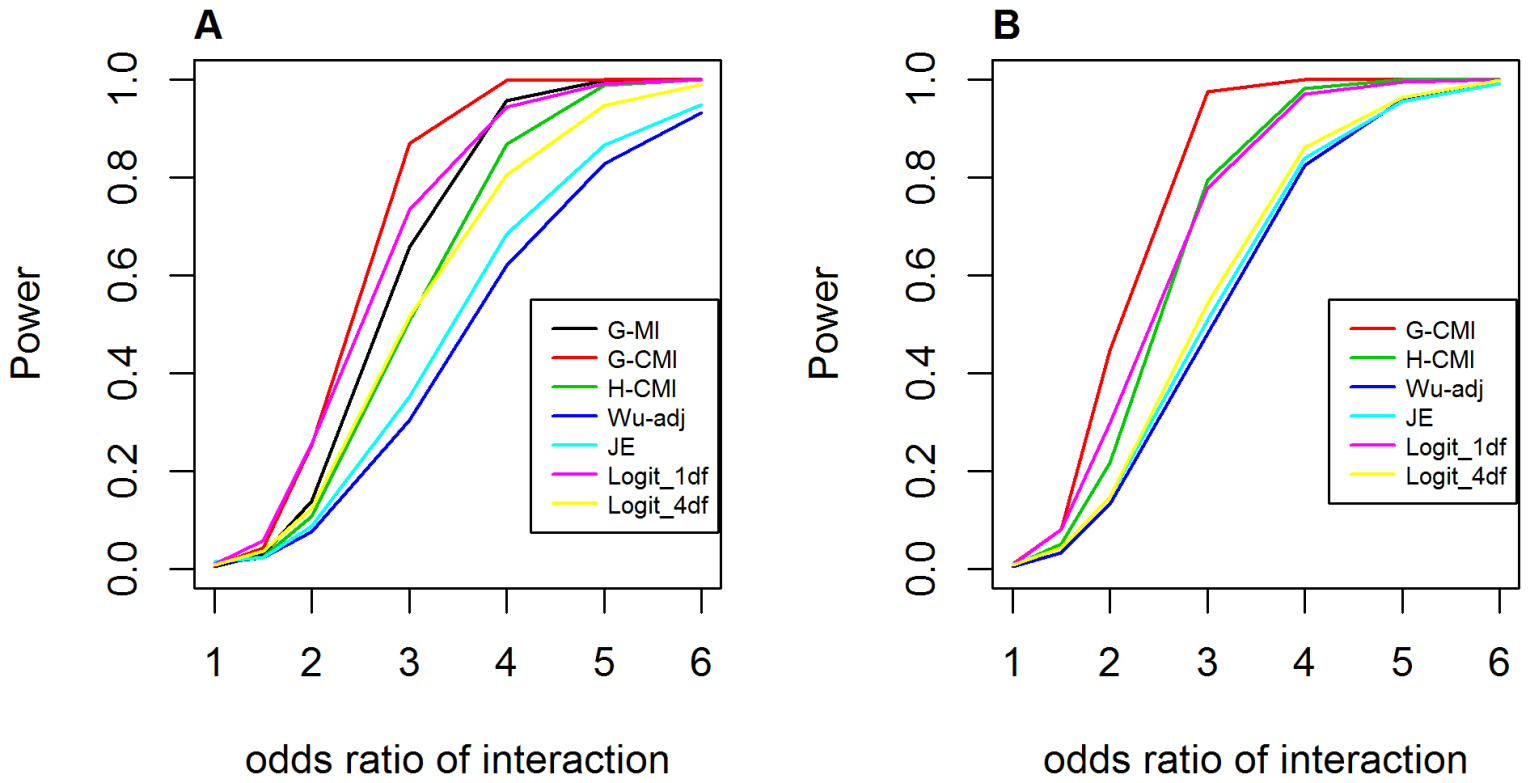
Power curves for testing interaction under the recessive-recessive interaction model. **A**. Assuming no main effect at both loci (*OR_G_* = *OR_H_* = 1.0); **B**. Assuming main effect at one locus (*OR_G_* = 2.0). G-MI: *GenoMI*; G-CMI: *GenoCMI*; H-CMI: *GameteCMI*; Wu-adj: Adjusted Wu statistics; JE: Joint Effects statistics; Logit_1 df: logistic regression model with 1 df test; Logit_4 df: logistic regression model with 4 df test. Disease prevalence was chosen at 0.02.

Figure 12 shows the power curves under the recessive-recessive interaction model. In the scenario of neither loci having main effect, *GenoCMI* appeared to have the best performance among all metrics or statistics, with >80% power at *OR_GH_* = 3.0 or above, followed by the logistic regression with 1 df test and *GameteCMI* in order. Interestingly, *GenoMI* performed relatively poor in this scenario. In the presence of main effect at one locus, *GenoCMI* again outperformed other approaches. *GameteCMI* seemed to have comparable performance to the logistic regression with 1 df test under the correct genetic model and better performance than the adjusted Wu statistic and the joint effect statistic as well.

### Application to Real Data Examples

To evaluate the utility of the entropy-based metrics in detecting gene-gene interactions in the real settings, we analyzed several published datasets. The first example was about the epistatic interaction between the hemoglobin (*Hb*) and *α* ^+^-thalassemia genes on protecting against malaria. The *S* variant of *Hb* gene, located on chromosome 11, is known to be responsible for sickle-cell disease. The *α* ^+^-thalassemia gene, located on chromosome 16, can cause the *α* ^+^-thalassemia if defective alleles are produced. Although the hemoglobin (*Hb*) and *α* ^+^-thalassemia genes can result in undesirable blood disorders, numerous evidence indicate a protective role for the mutant genes against the more severe manifestations of malaria. However, the mechanisms underlying this protection are poorly understood, in particular much less is known about the *α* ^+^-thalassemia gene and how it exerts anti-malaria effects when inherited in combination with the *Hb* gene. Here we analyzed a datum from a birth cohort study of the incidence of hospital admission with malaria and severe malaria from Kilifi District Hospital on the coast of Kenya in Africa [34]. In this cohort, 2104 children were genotyped. The *Hb* gene was genotyped as *HbAA* and *HbAS*, representing the wild type and mutant type, respectively The wild-type homozygote, heterozygote and mutant homozygote genotypes of the *α* ^+^-thalassemia gene were encoded as *αα/αα*, −*α/αα*, and −*α/−α*, respectively. For comparison, the logistic regression models based on different genotypic coding (corresponding to the dominant-dominant model and additive-additive model, respectively), and based on coding the *Hb* and *α* ^+^-thalassemia gene as dummy variables, were also provided. The results of three entropy-based metrics, including our proposed two CMI-based metrics, are summarized in Table 3. For comparison, Table 3 also lists the *P*-values obtained by using the Poisson regression, originally reported by Williams et al. [34], and *P*-values obtained by using logistic regression with 1 df test and 4 df test, respectively. Our results showed that *P*-values obtained by using *GenoMI*, *GenoCMI* and *GameteCMI* were smaller than those obtained by using Poisson regression analysis. *GameteCMI* achieved lower *P*-values compared to logistic regression with either 1 df test or 4 df test, whereas *P*-values obtained by using *GenoCMI* were comparably higher than those obtained by using the two logistic regression based tests.

**Table 3:** Comparison of *P*-values in testing gene-gene interaction between hemoglobin (*Hb*) gene and *α* ^+^-thalassemia gene.

	Genotypes		Frequency^a^	*P*-values obtained by					
				*GenoMI*	*GenoCMI*	*GameteCMI*	Wald test^b^	*OR* c	*OR* d
Malaria Admission	*HbAA*	*αα*/*αα*	168/458	1.84E-10	3.88E-3	3.30E-5	2.60E-2	4.35E-4	8.63E-4
	*HbAA*	*−α*/*αα*	187/680						
	*HbAA*	*−α*/*−α*	56/246						
	*HbAs*	*αα*/*αα*	6/107						
	*HbAs*	*−α*/*αα*	9/141						
	*HbAs*	*−α*/*−α*	10/36						
Severe Malaria	*HbAA*	*αα*/*αα*	67/559	2.12E-7	1.17E-3	2.24E-6	1.20E-3	1.40E-5	1.80E-4
	*HbAA*	*−α*/*αα*	53/814						
	*HbAA*	*−α*/*−α*	17/285						
	*HbAs*	*αα*/*αα*	0/113						
	*HbAs*	*−α*/*αα*	2/148						
	*HbAs*	*−α*/*−α*	5/41						

a frequencies were shown as No. of case/No. of control.

b P-values reported by Williams et al.

c the lowest *P*-value among logistic regression models by assuming additive × additive, dominant × dominant and recessive × recessive interaction models, respectively.

d obtained by logistic regression model by coding genotypes as factors.

The second example was composed of several studies concerning the interaction between SNP309 in *MDM2* gene (*MDM2 309T*>*G*, located on chromosome 12) and codon72 polymorphism in *p53* gene (*p53 72Arg*>*Pro*, located on chromosome 17) predisposing to different cancers. The *p53* gene encodes a tumor suppressor protein (*p53*) that is involved in cell cycle regulation as a trans-activator and acts as a tumor suppressor in many cancers. The mutant *p53 72Arg*>*Pro* is credited with a more efficient transcriptional activation and suppression of cell growth [35]. The *MDM2* gene is a target gene of the TP53 protein, meanwhile, an important negative regulator of the *p53* tumor suppressor. The mutant of *MDM2 309T*>*G* is proposed to elevate the expression of the product of *MDM2*, which binds the *p53* protein and targets its proteasomal degradation, thereby attenuating its response and diminishing its tumor suppressor function [36]. Investigation of the *p53*-*MDM2* interaction could provide valuable insight into the mechanisms underlying tumor oncogenesis afforded by these genes. In this example, we evaluated four phenotypes in three different populations, including gastric cardia cancer in Han population[37], lung cancer in Han population[38], hepatocellular carcinoma in patients infected with HBV in Korean population [39], and breast cancer nested in Nurse Health Study and Nurses' Health Study II [40]. The variants in *p53* and *MDM2* are *Pro* and *G*, respectively. Table 4 shows the *P*-values obtained by using three entropy-based metrics including two proposed CMI-based metrics. The *P*-values from the two logistic regression methods with either 1 df test or 4 df test were used for comparison. The *P*-values of *GameteCMI* in all the four cancer phenotypes were smaller than that of logistic regression analyses, while the *P*-values of *GenoCMI* were smaller than or similar to those of logistic regression with 1 df test and were smaller than those obtained by using logistic regression with 4 df tests. In conclusion, these two real data applications demonstrated that *GameteCMI* and *GenoCMI* achieved optimal or nearly optimal performance compared to the benchmark logistic regression based test.

**Table 4:** Application of entropy-based statistics for testing gene-gene interaction between SNP309 in *MDM2* gene and codon72 polymorphism in *p53* gene.

p53 72Arg>Pro	GCC		LC		HCC		BC	
	Case^a^	Control^b^	Case^a^	Control^b^	Case^a^	Control^b^	Case^a^	Control^b^
Arg/Arg	19/59/45	96/162/58	62/170/89	122/223/80	23/48/39	40/56/28	349/317/104	488/539/166
Arg/Pro	61/119/65	150/222/114	127/259/120	222/343/166	18/58/35	38/53/45	218/266/63	346/365/92
Pro/Pro	27/72/33	52/114/32	60/132/87	74/145/45	4/19/43	6/23/8	40/45/14	60/52/10
GenoMI	3.88E-06		5.11E-08		9.34E-07		2.02E-01	
GenoCMI	1.36E-03		4.99E-02		5.69E-05		7.41E-02	
GameteCMI	1.90E-03		1.28E-03		2.44E-04		1.28E-03	
OR_A_ 1	9.46E-02		9.90E-01		8.49E-02		1.87E-02	
OR_D_ 2	7.99E-02		2.96E-01		4.90E-01		1.42E-02	
OR_R_ 3	7.45E-01		1.48E-02		7.15E-04		1.56E-01	
OR_C_ 4	7.92E-02		2.26E-02		3.92E-03		9.08E-02	

a frequencies were shown as No. of individuals genotyped as *TT*/*TG*/*GG* of *MDM2 309T*>*G* in case.

b frequencies were shown as No. of individuals genotyped as *TT*/*TG*/*GG* of *MDM2 309T*>*G* in control.

1 obtained by logistic regression model assuming additive × additive model.

2 obtained by logistic regression model assuming dominant × dominant model.

3 obtained by logistic regression model assuming recessive × recessive model.

4 obtained by logistic regression model by coding genotypes as factors.

GCC: gaster cardia cancer; LC: lung cancer; HCC: hepatacelluar cancer; BC: breast cancer.

## Discussion

In this study, we attempted to solve the potential false positive problem associated with current model-free or data mining approaches for genome-wide interaction analysis, by using the entropy-based metrics as examples. Inspired by the work of Ueki and Cordell [27] who studied the similar issue associated with model-based approaches, we first mathematically and empirically demonstrated that the widely used mutual information based metric (*GenoMI*) was not able to distinguish the genuine interaction effect from the joint effect. In particular, when the main effects at two loci were large, the inflation of type 1 error in testing interaction effect was much salient. To remedy this issue, we proposed to use two conditional mutual information (CMI) based metrics (named as *GenoCMI* and *GameteCMI*, respectively). By mathematical derivations and extensive simulations, we showed that the two CMI-based metrics worked well to control false positive error in various two-locus interaction models.

To evaluate the capability of the proposed CMI-based metrics to control the type 1 error, we considered varieties of parameter settings in a systematic way. In absence of both main effect and interaction effect or presence of main effect at one locus, the proposed two CMI-based metrics achieved adequate control of type 1 error in both common and rare disease models, with only feebly influenced by genetic models and the sizes of main effect. In the scenario of a common disease and both loci having main effects, *GenoCMI* and the previous joint effect statistic could control false positive error well under the recessive-recessive or dominant-dominant genetic models, whereas *GameteCMI* as well as the adjusted Wu statistic exhibited noticeable inflated type 1 error under the dominant-dominant genetic model. Unfortunately, we are not very clear about why the GameteCMI appeared having an inconsistent type 1 error rate under dominant-dominant interaction models. Although this phenomenon was also observed (but without explanations) in the previous studies (e.g. the previously proposed adjusted Wu statistic that is in essence a haplotype-based statistic, see [27]), we have not found a good way to adjust for this bias yet. In the scenarios of additive-additive genetic model, except for the benchmark logistic regression models, none of the remaining six metrics or statistics could well control the false positive error, when the main effects were large (e.g. *OR*≥3.0). Fortunately, both our proposed CMI-based metrics and several previous statistics (adjusted Wu statistics and the joint effect statistic) were able to control type 1 error with moderate main effects at both loci, e.g. *OR* = 2.0, which is often the case for most complex diseases. Furthermore, based on our simulation studies, the disease prevalence (*K*) and case/control ratio (*P_A_*), two important factors underlying a case-control study, appeared to have subtle influence on the control of type 1 error.

In terms of statistical power for detecting gene-gene interaction, the proposed CMI-based metrics in general had better performance than the adjusted Wu statistic and the joint effect statistic, and had comparable performance to the benchmark logistic regression. In the absence of main effect at both loci, the *GenoMI* performed best in detecting the additive-additive or dominant-dominant interaction effect. However, this advantage was somewhat tempered by its possibility of inflated type 1 error. The trade-off between the gain of power and the risk of false positive error becomes a difficult balance to strike in real data analysis. One feasible consideration is to apply multistage tests or an ensemble approach, in which we first use *GenoMI* to filter out non-informative gene pairs, followed by refining gene pairs that are with genuine interaction effects by using *GenoCMI* and *GameteCMI* or other alternatives mentioned in this study.

Finally, we should recognized that several issues still remained unattended in this study, although we have very carefully explored the statistical properties of the two proposed CMI-based metrics for detecting gene-gene interaction. First, we did not concern varieties of definitions of interaction, which can be very diverse from statistical standpoints to biological ones [41], for examples, from statistically defined as deviation from additive effects [42], [43] or compositional epistasis [44], [45] to various forms of biological interactions such as physical binding of proteins [46] and transcriptional regulations. In this study, we mainly concerned in detecting the statistical interactions, and whether these proposed information-based metrics are able to reveal various biological mechanisms remains unclear. Second, we only considered several common two-locus interaction models. Theoretically, the number of two-locus models for binary complex human diseases is up to 50 models under full penetrance assumption [47] or 69 models for continuous penetrance [48]. A natural extension should comprehensively examine their capability of the newly proposed metrics in detecting genuine interaction under various two-locus models. Because of their model-free nature, the two proposed CMI-based metrics are anticipated to be robust to various forms of interactions, which, however, requires further studies to verify. Third, we only considered the simplest case of two independent SNP loci (i.e. located on different chromosomes) in this study. The more complicated scenarios involve, for example, the multiple SNP loci from the same LD block or correlation of the tests due to the sharing of the same SNP etc. To deal with these scenarios requires a more involved method to distinguish the true epistasis from other reasons. Anyway, given their potential of capturing various forms of non-linear interactions, the newly proposed CMI-based metrics could become promising and powerful alternatives for detecting large-scale interactions.

## Supporting Information

Figure S1 **Chi-squared Q-Q plots for the dominant model with main effect at one locus (Schema 2).** Top panels: **A**. *GenoMI*; **B**. *GenoCMI*; **C**. *GameteCMI*. Middle panels: **D**. original Wu et al statistic; **E**. adjusted Wu statistic; **F**. joint effect statistic. Bottom panel: **G**. logistic regression model with 1 df test; **H**. logistic regression model with 4 df test.
(TIFF)

Figure S2 **Chi-squared Q-Q plots for the additive model with main effect at one locus (Schema 2).** Top panels: **A**. *GenoMI*; **B**. *GenoCMI*; **C**. *GameteCMI*. Middle panels: **D**. original Wu et al statistic; **E**. adjusted Wu statistic; **F**. joint effect statistic. Bottom panel: **G**. logistic regression model with 1 df test; **H**. logistic regression model with 4 df test.
(TIFF)

Figure S3 **Chi-squared Q-Q plots for the dominant-dominant model with main effect at one locus, when disease prevalence varied (Schema 4).** Assuming a main effect at single locus (ORG = 2.0) and 1∶1 case/control ratio. Top panels: **A.** GenoMI; **B.** GenoCMI; **C.** GameteCMI. Middle panels: **D.** original Wu et al statistic; **E.** adjusted Wu statistic; **F.** joint effect statistic. Bottom panel: **G.** logistic regression model with 1 df test; **H.** logistic regression model with 4 df test.
(TIFF)

Figure S4 **Chi-squared Q-Q plots for the additive-additive model with main effect at one locus, when disease prevalence varied (Schema 4).** Assuming a main effect at single locus (ORG = 2.0) and 1∶1 case/control ratio. Top panels: **A.** GenoMI; **B.** GenoCMI; **C.** GameteCMI. Middle panels: **D.** original Wu et al statistic; **E.** adjusted Wu statistic; **F.** joint effect statistic. Bottom panel: **G.** logistic regression model with 1 df test; **H.** logistic regression model with 4 df test.
(TIFF)

Figure S5 **Chi-squared Q-Q plots for the dominant-dominant model with main effects at both loci, when disease prevalence varied (Schema 5).** Assuming main effects at both locus (*OR_G_* = *OR_H_* = 2.0) and 1∶1 case/control ratio. Top panels: **A**. *GenoMI*; **B**. *GenoCMI*; **C**. *GameteCMI*. Middle panels: **D**. original Wu et al statistic; **E**. adjusted Wu statistic; **F**. joint effect statistic. Bottom panel: **G**. logistic regression model with 1 df test; **H**. logistic regression model with 4 df test
(TIFF)

Figure S6 **Chi-squared Q-Q plots for the additive-additive model with main effects at both loci, when disease prevalence varied (Schema 5).** Assuming main effects at both locus (*OR_G_* = *OR_H_* = 2.0) and 1∶1 case/control ratio. Top panels: **A**. *GenoMI*; **B**. *GenoCMI*; **C**. *GameteCMI*. Middle panels: **D**. original Wu et al statistic; **E**. adjusted Wu statistic; **F**. joint effect statistic. Bottom panel: **G**. logistic regression model with 1 df test; **H**. logistic regression model with 4 df test.
(TIFF)

Figure S7 **Chi-squared Q-Q plots for the dominant-dominant model with main effects at both loci, when case/control ratios varied (Schema 7).** Assuming main effects at both locus (*OR_G_* = *OR_H_* = 2.0) and disease prevalence 0.02. Top panels: **A**. *GenoMI*; **B**. *GenoCMI*; **C**. *GameteCMI*. Middle panels: **D**. original Wu et al statistic; **E**. adjusted Wu statistic; **F**. joint effect statistic. Bottom panel: **G**. logistic regression model with 1 df test; **H**. logistic regression model with 4 df test.
(TIFF)

Figure S8 **Chi-squared Q-Q plots for the additive-additive model with main effects at both loci, when case/control ratios varied (Schema 7).** Assuming main effects at both locus (*OR_G_* = *OR_H_* = 2.0) and disease prevalence 0.02. Top panels: **A**. *GenoMI*; **B**. *GenoCMI*; **C**. *GameteCMI*. Middle panels: **D**. original Wu et al statistic; **E**. adjusted Wu statistic; **F**. joint effect statistic. Bottom panel: **G**. logistic regression model with 1 df test; **H**. logistic regression model with 4 df test.
(TIFF)

Text S1 **Mathematical derivation of** ***GenoCMI*** **and** ***GameteCMI*** **metrics.**
(DOC)

Text S2 **Mathematical derivation of asymptotic distributions of** ***GenoCMI*** **and** ***GameteCMI*** **metrics.**
(DOC)
