# Supplementary figures and images for: To Control False Positives in Gene-Gene Interaction Analysis: Two Novel Conditional Entropy-Based Approaches
Source: PLoS One. 2013 Dec 10;8(12):e81984. doi: 10.1371/journal.pone.0081984 (PMC3858311; doi:10.1371/journal.pone.0081984)

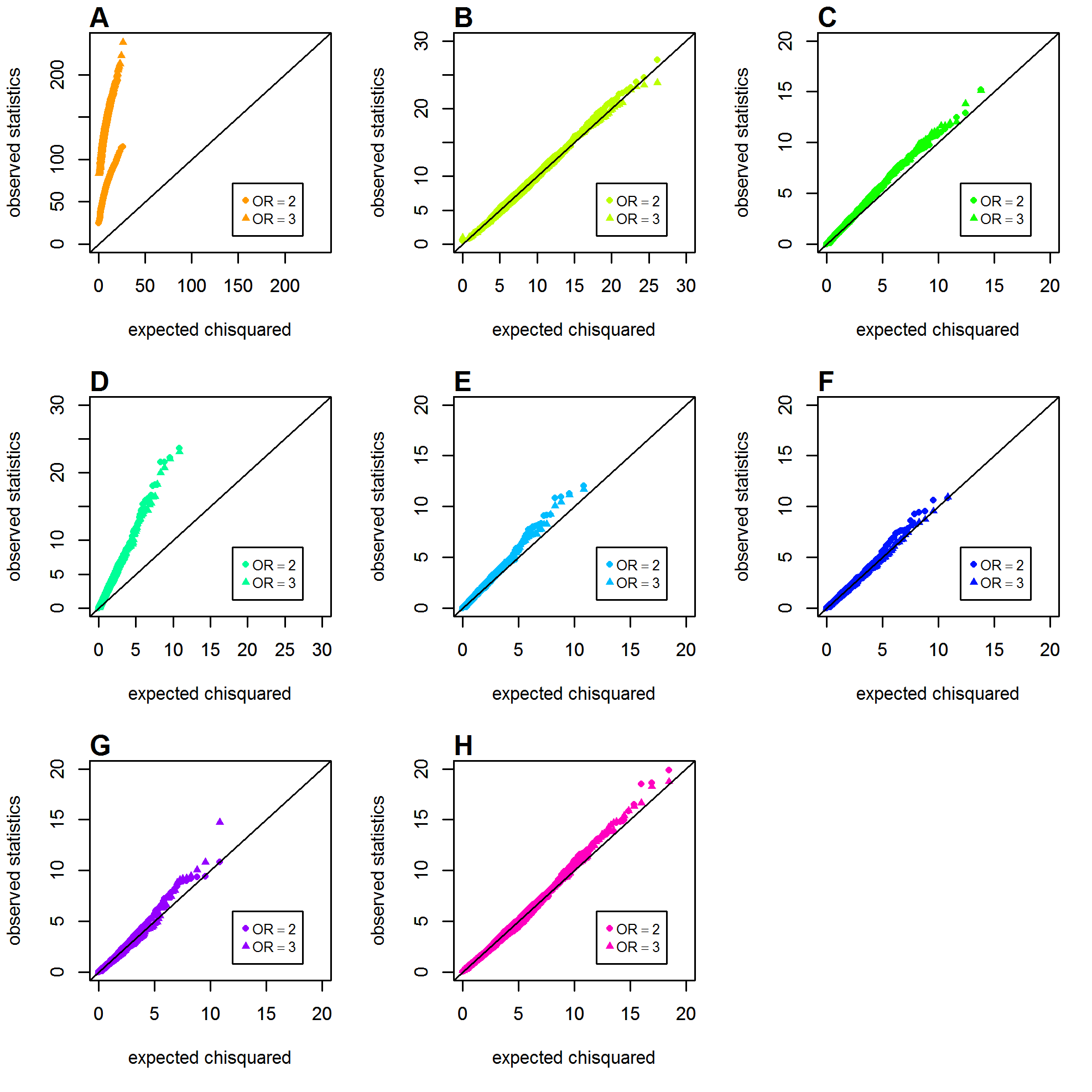

Supplement: Figure S1 — Chi-squared Q-Q plots for the dominant model with main effect at one locus (Schema 2). Top panels: A. GenoMI; B. GenoCMI; C. GameteCMI. Middle panels: D. original Wu et al statistic; E. adjusted Wu statistic; F. joint effect statistic. Bottom panel: G. logistic regression model with 1 df test; H. logistic regression model with 4 df test. (TIFF) [file pone.0081984.s001.tiff]

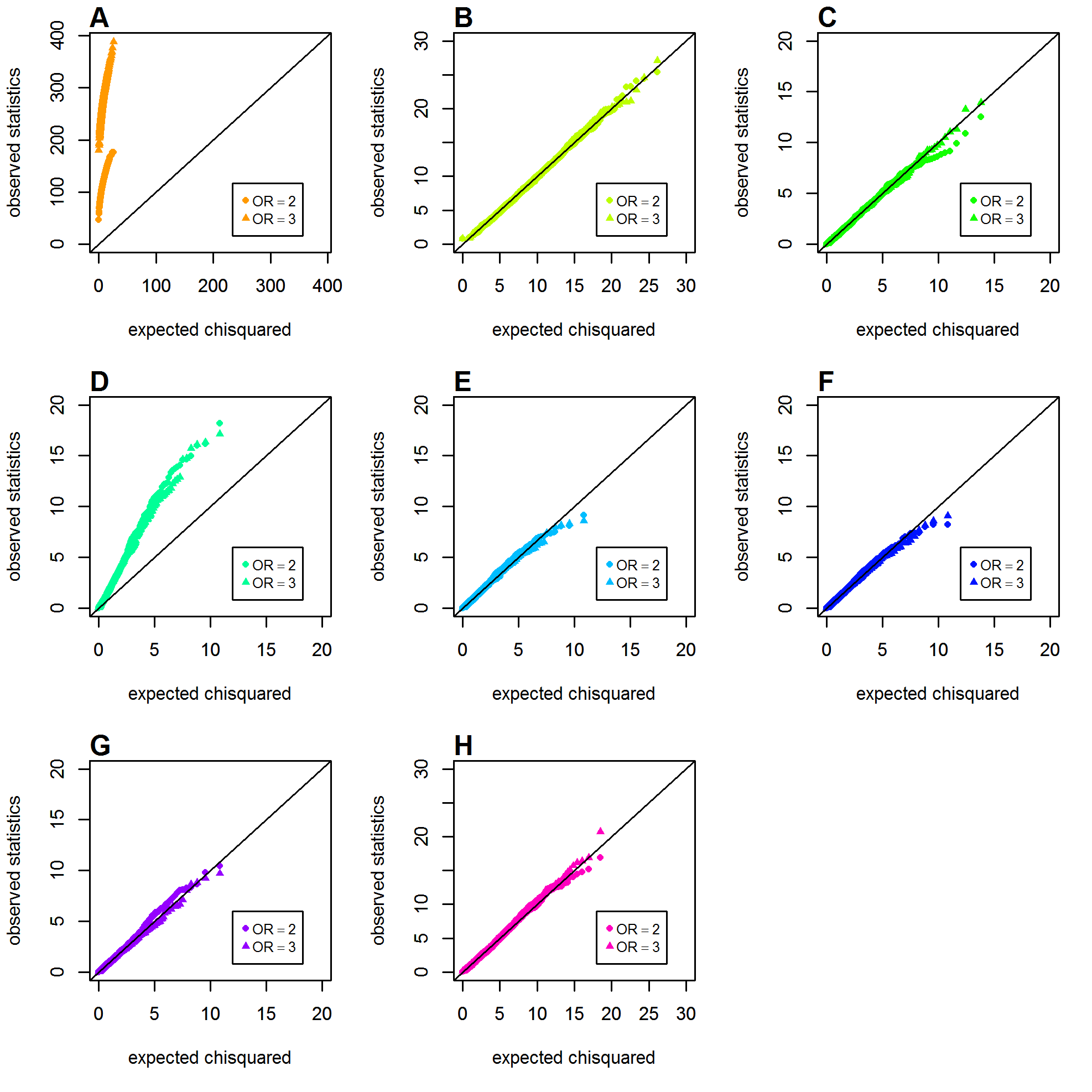

Supplement: Figure S2 — Chi-squared Q-Q plots for the additive model with main effect at one locus (Schema 2). Top panels: A. GenoMI; B. GenoCMI; C. GameteCMI. Middle panels: D. original Wu et al statistic; E. adjusted Wu statistic; F. joint effect statistic. Bottom panel: G. logistic regression model with 1 df test; H. logistic regression model with 4 df test. (TIFF) [file pone.0081984.s002.tiff]

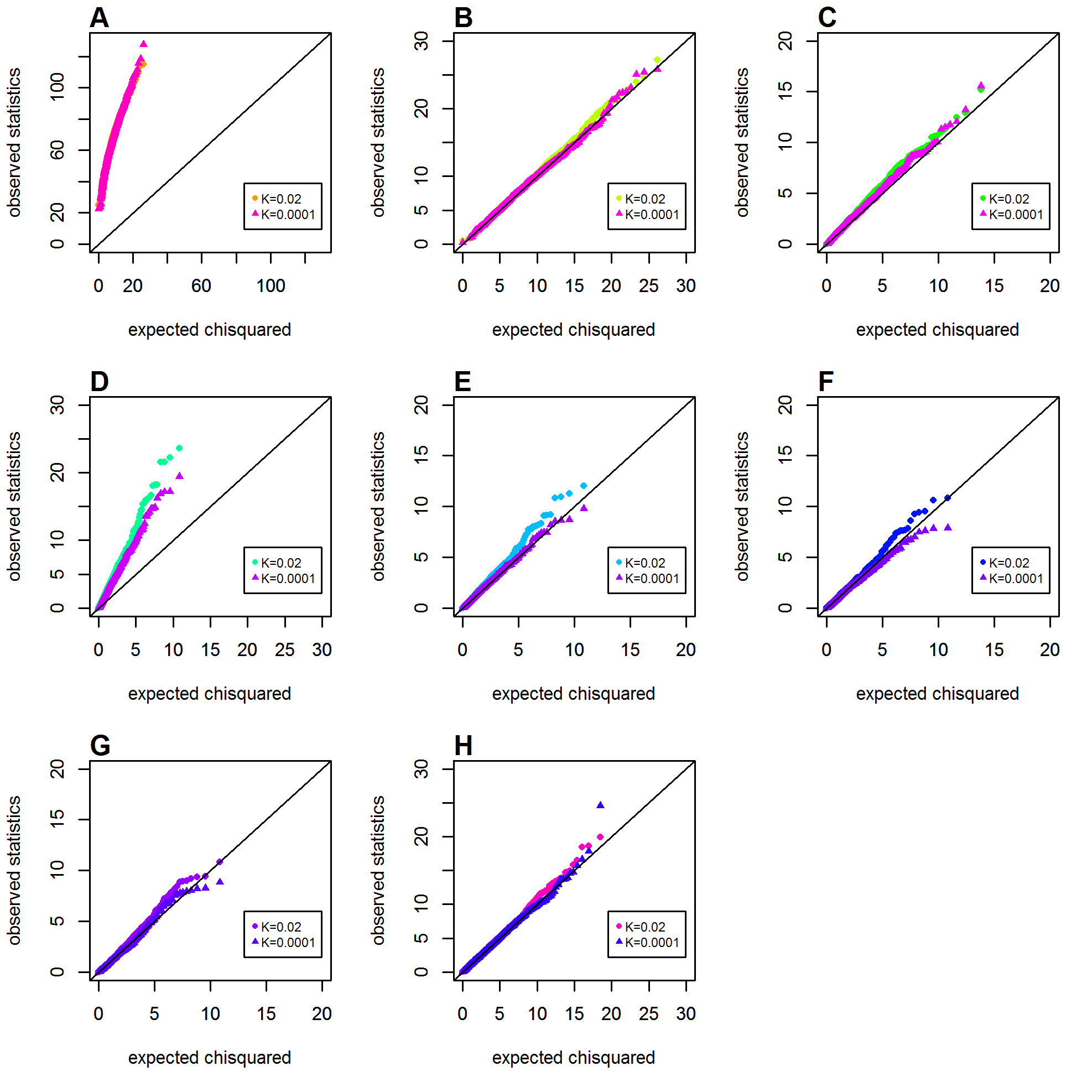

Supplement: Figure S3 — Chi-squared Q-Q plots for the dominant-dominant model with main effect at one locus, when disease prevalence varied (Schema 4). Assuming a main effect at single locus (ORG = 2.0) and 1∶1 case/control ratio. Top panels: A. GenoMI; B. GenoCMI; C. GameteCMI. Middle panels: D. original Wu et al statistic; E. adjusted Wu statistic; F. joint effect statistic. Bottom panel: G. logistic regression model with 1 df test; H. logistic regression model with 4 df test. (TIFF) [file pone.0081984.s003.tiff]

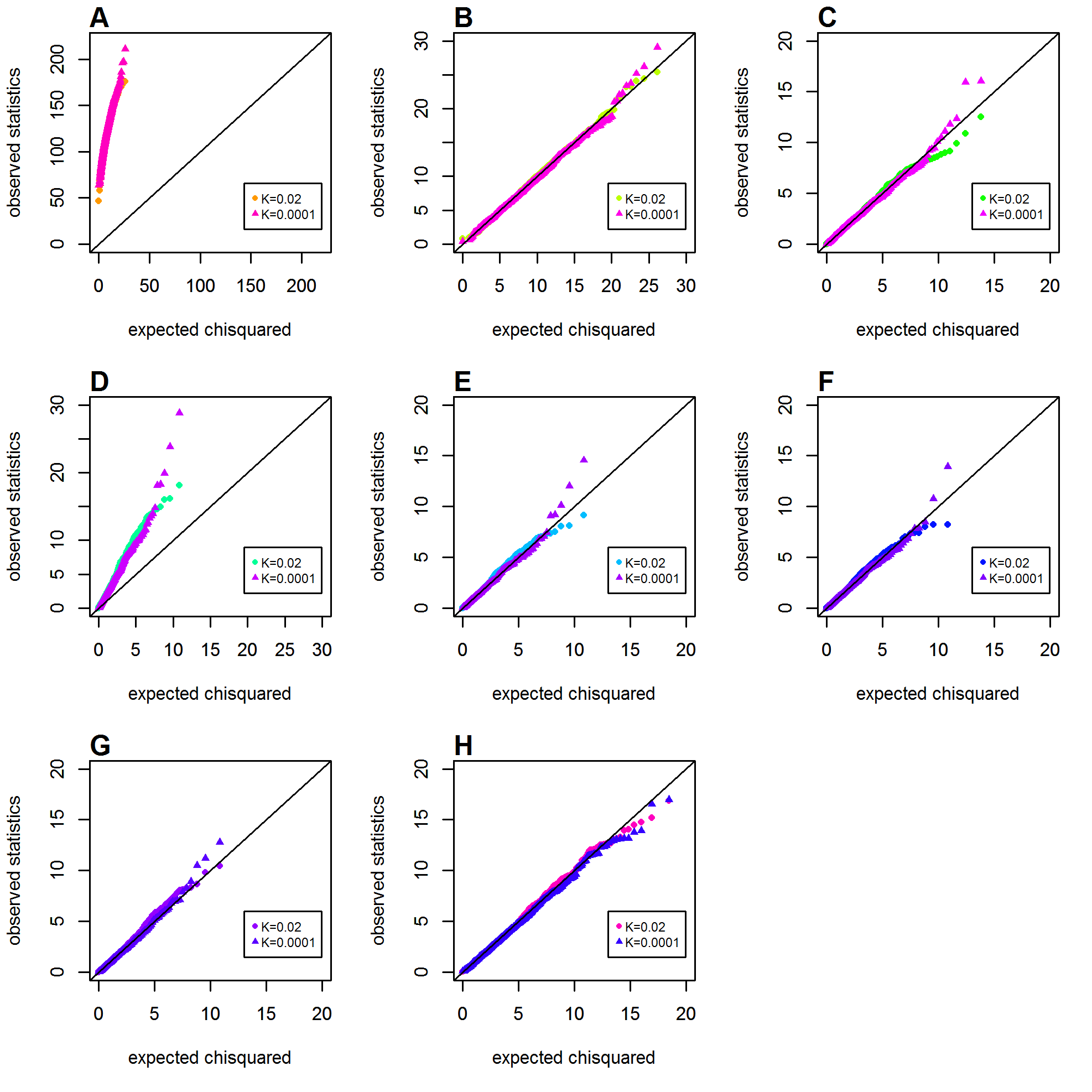

Supplement: Figure S4 — Chi-squared Q-Q plots for the additive-additive model with main effect at one locus, when disease prevalence varied (Schema 4). Assuming a main effect at single locus (ORG = 2.0) and 1∶1 case/control ratio. Top panels: A. GenoMI; B. GenoCMI; C. GameteCMI. Middle panels: D. original Wu et al statistic; E. adjusted Wu statistic; F. joint effect statistic. Bottom panel: G. logistic regression model with 1 df test; H. logistic regression model with 4 df test. (TIFF) [file pone.0081984.s004.tiff]

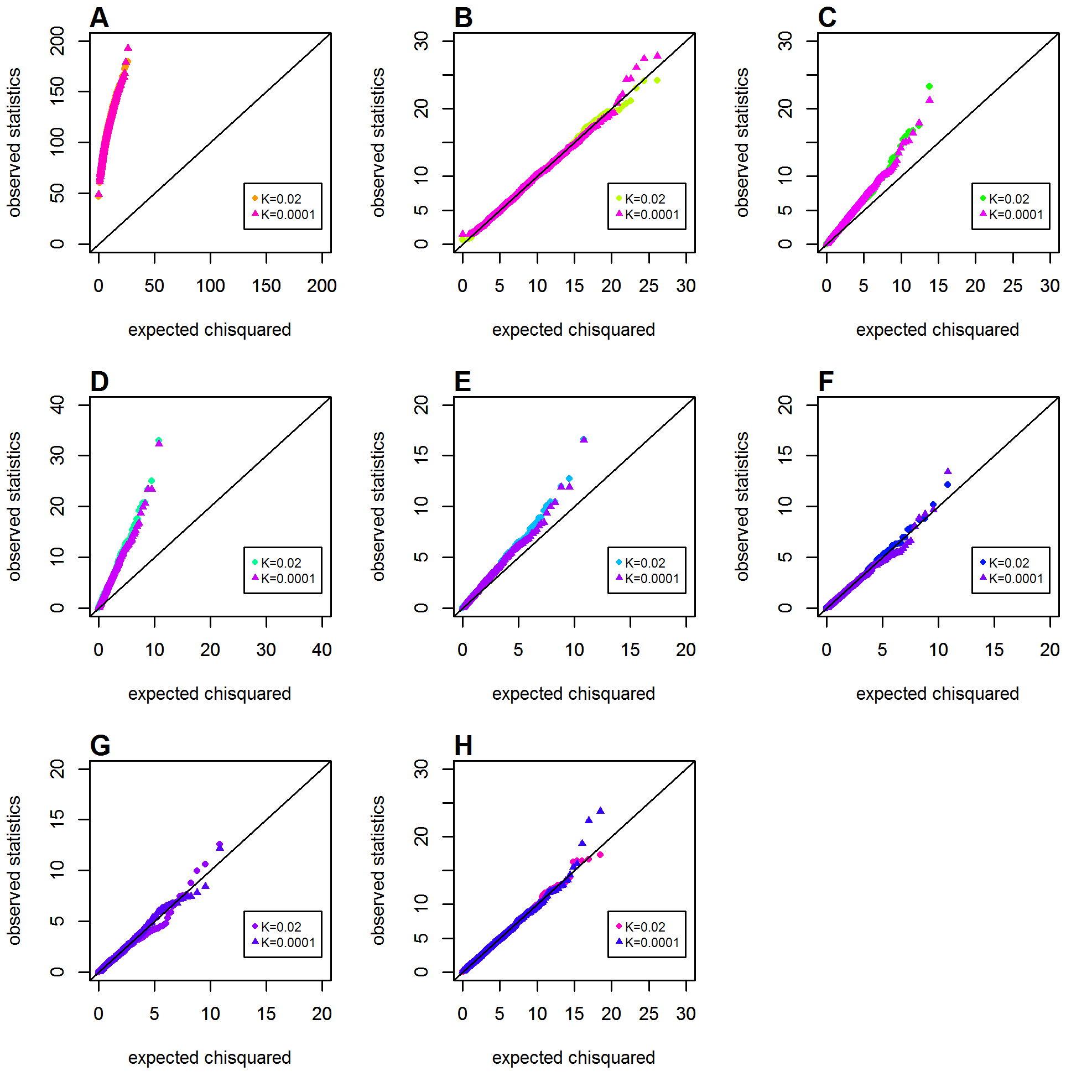

Supplement: Figure S5 — Chi-squared Q-Q plots for the dominant-dominant model with main effects at both loci, when disease prevalence varied (Schema 5). Assuming main effects at both locus (ORG = ORH = 2.0) and 1∶1 case/control ratio. Top panels: A. GenoMI; B. GenoCMI; C. GameteCMI. Middle panels: D. original Wu et al statistic; E. adjusted Wu statistic; F. joint effect statistic. Bottom panel: G. logistic regression model with 1 df test; H. logistic regression model with 4 df test (TIFF) [file pone.0081984.s005.tiff]

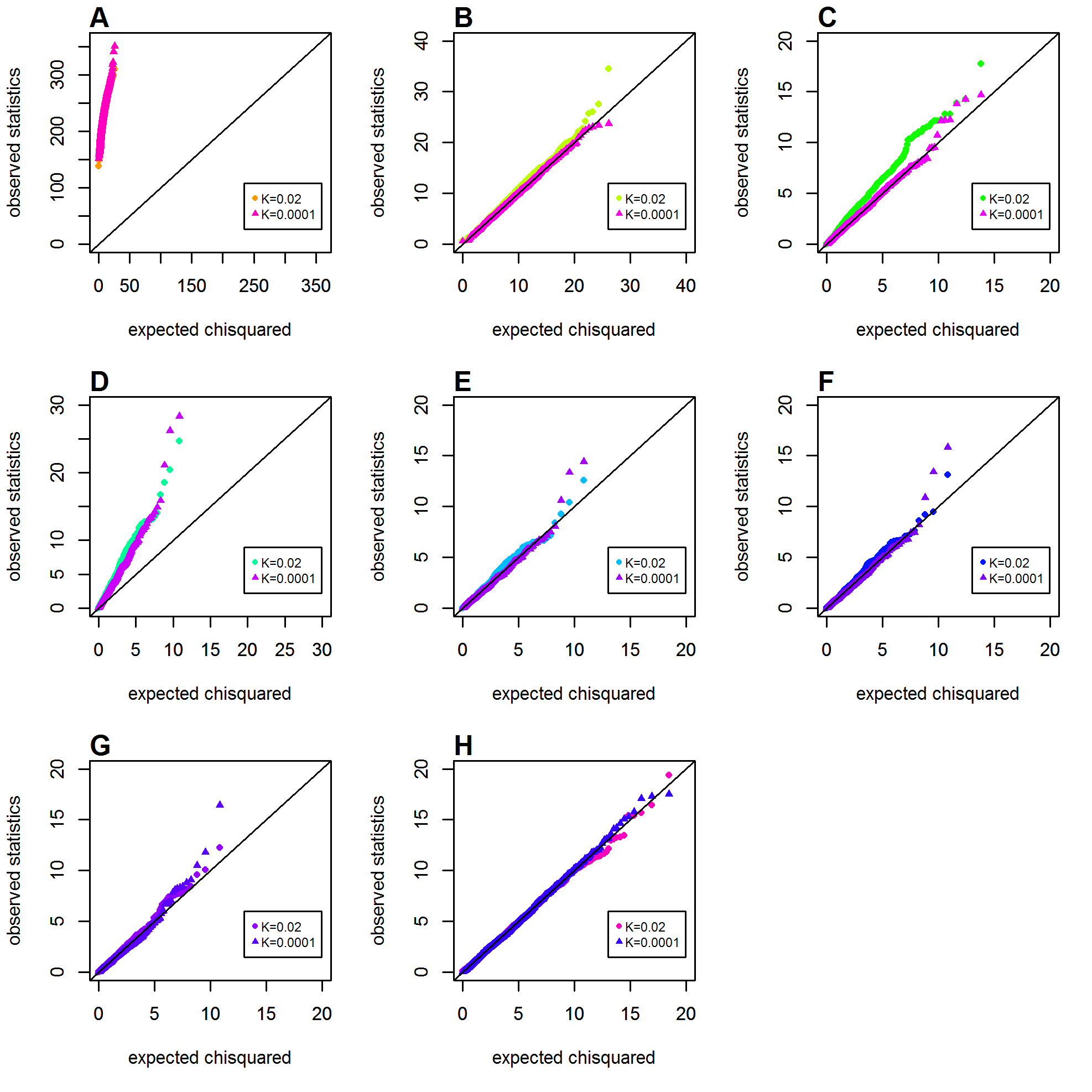

Supplement: Figure S6 — Chi-squared Q-Q plots for the additive-additive model with main effects at both loci, when disease prevalence varied (Schema 5). Assuming main effects at both locus (ORG = ORH = 2.0) and 1∶1 case/control ratio. Top panels: A. GenoMI; B. GenoCMI; C. GameteCMI. Middle panels: D. original Wu et al statistic; E. adjusted Wu statistic; F. joint effect statistic. Bottom panel: G. logistic regression model with 1 df test; H. logistic regression model with 4 df test. (TIFF) [file pone.0081984.s006.tiff]

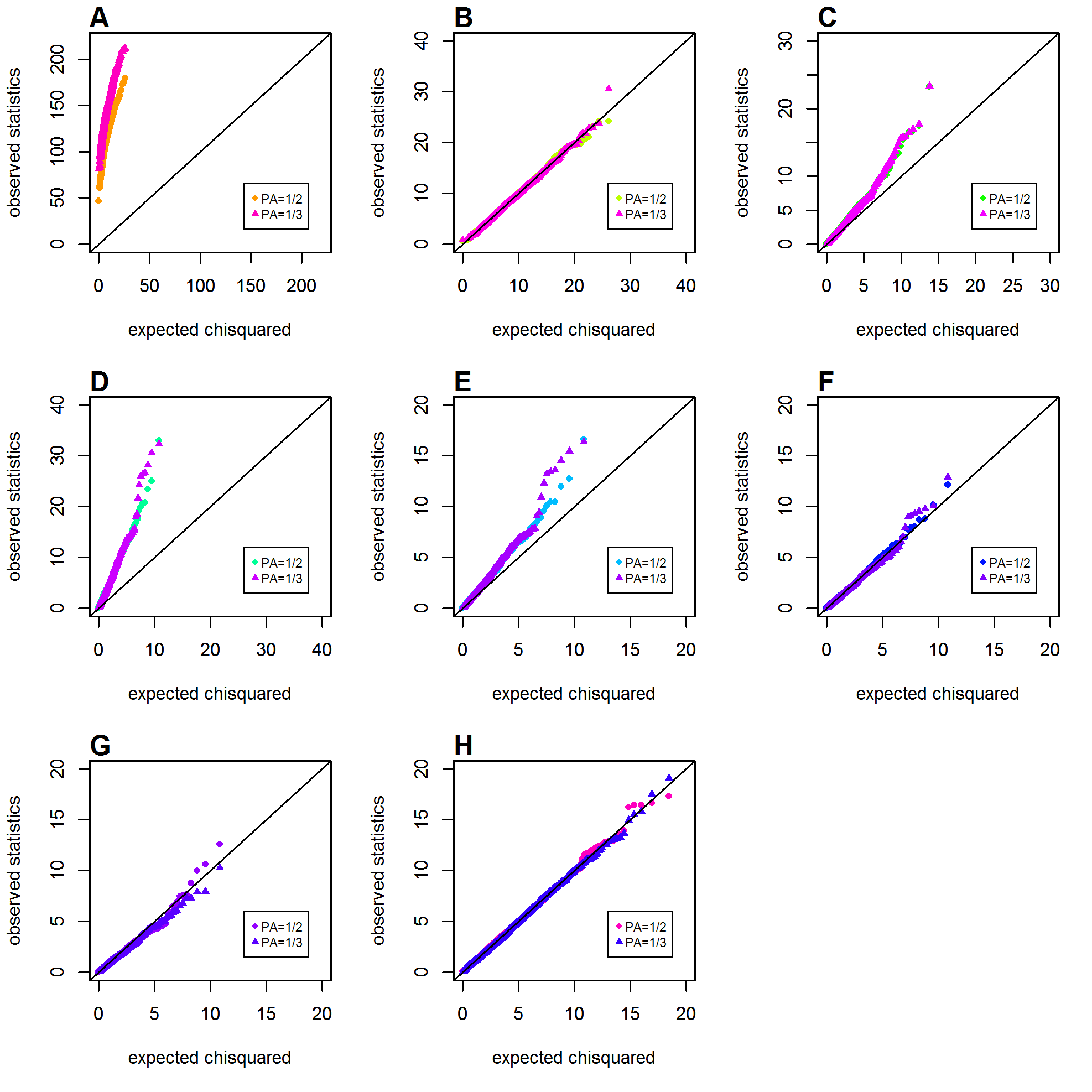

Supplement: Figure S7 — Chi-squared Q-Q plots for the dominant-dominant model with main effects at both loci, when case/control ratios varied (Schema 7). Assuming main effects at both locus (ORG = ORH = 2.0) and disease prevalence 0.02. Top panels: A. GenoMI; B. GenoCMI; C. GameteCMI. Middle panels: D. original Wu et al statistic; E. adjusted Wu statistic; F. joint effect statistic. Bottom panel: G. logistic regression model with 1 df test; H. logistic regression model with 4 df test. (TIFF) [file pone.0081984.s007.tiff]

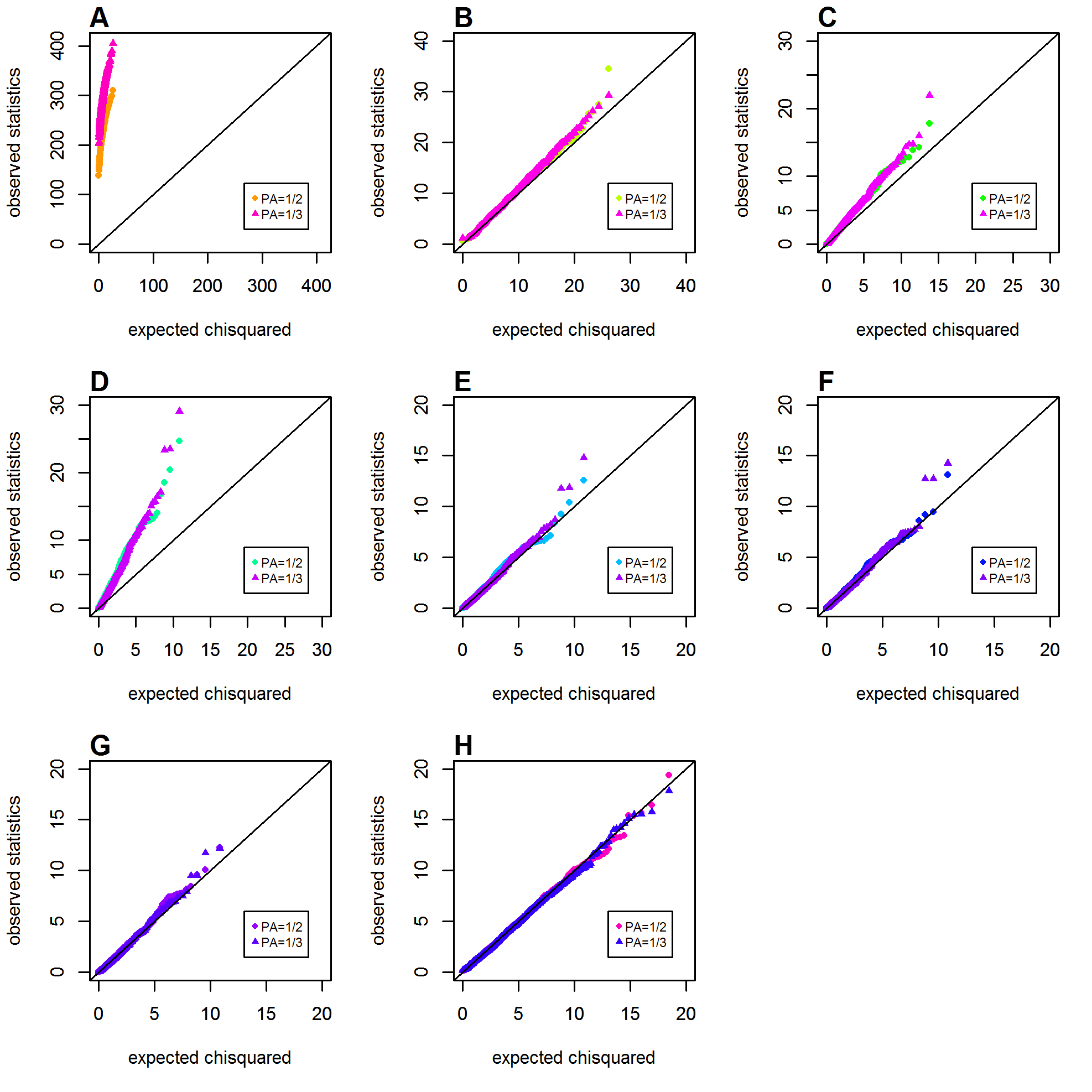

Supplement: Figure S8 — Chi-squared Q-Q plots for the additive-additive model with main effects at both loci, when case/control ratios varied (Schema 7). Assuming main effects at both locus (ORG = ORH = 2.0) and disease prevalence 0.02. Top panels: A. GenoMI; B. GenoCMI; C. GameteCMI. Middle panels: D. original Wu et al statistic; E. adjusted Wu statistic; F. joint effect statistic. Bottom panel: G. logistic regression model with 1 df test; H. logistic regression model with 4 df test. (TIFF) [file pone.0081984.s008.tiff]
