# Supplementary material for: To Control False Positives in Gene-Gene Interaction Analysis: Two Novel Conditional Entropy-Based Approaches
Source: PLoS One. 2013 Dec 10;8(12):e81984. doi: 10.1371/journal.pone.0081984 (PMC3858311; doi:10.1371/journal.pone.0081984)
Supplement: Text S1 — Mathematical derivation of GenoCMI and GameteCMI metrics. (DOC) [file pone.0081984.s009.doc]

**Text S1 Mathematical derivation of *GenoCMI* and *GameteCMI* metrics**

**Conditional mutual information based on genotype (*GenoCMI*)**

For convenience, consider two unlinked diallelic loci, *G* and *H*, with locus *G* having alleles *a* and *A* and locus *H* having alleles *b* and *B*. Let alleles *A* and *B* denote the risk allele of loci *G* and *H*, respectively. Let *i* and *j* (*i*, *j* =0, 1 or 2) represent the genotypes of loci *G* and *H*, respectively, where 0, 1 and 2 denote the number of risk alleles in a genotype (that is, wild-type homozygote, heterozygote and mutant homozygote, respectively). Let *D* denote the disease status of each individual, where *D*=1 (*D*=0) indicates affected (unaffected). The conditional mutual information based on genotype, denoted as *GenoCMI*, could be defined and formulated as following:

$$GenoCMI=\sum_{d} \sum_{i} \sum_{j} P(G=i,H=j,D=d)\log\left(\frac{P(G=i,H=j\left|D=d\right.)}{P(G=i\left|D=d\right.)P(H=j\left|D=d\right.)}\right)$$

$=\sum_{i} \sum_{j} \left[P_{A}P_{ij}^{A}\log\left(\frac{P_{ij}^{A}}{P_{i\cdot}^{A}P_{\cdot j}^{A}}\right)+P_{N}P_{ij}^{N}\log\left(\frac{P_{ij}^{N}}{P_{i\cdot}^{N}P_{\cdot j}^{N}}\right)\right]$ (1)

where $P_{ij}^{A}$($P_{ij}^{N}$), $P_{i\cdot}^{A}$($P_{i\cdot}^{N}$), and $P_{\cdot j}^{A}$($P_{\cdot j}^{N}$) indicate the joint genotype frequency of loci *G* and *H*, the genotype frequency of locus *G*, and the genotype frequency of locus *H* in affected (unaffected) population, respectively (i.e. $P_{11}^{A}$, $P_{1\cdot}^{A}$ and $P_{\cdot1}^{A}$ indicate the frequencies of *AaBb*, *Aa* and *Bb* in affected population, respectively). $P_{A}$($P_{N}$) is the frequencies of affected (unaffected) people in the combined population (in fact, $P_{N}=1-P_{A}$). In a case-control study, it is the proportion of cases (controls), and in a cohort study, it is the estimated prevalence (1-prevalence) in the general population.

Let$f_{ij}$, $f_{i\cdot}$ and $f_{\cdot j}$ be measures of penetrance of joint genotype *ij* of loci *G* and *H*, genotype *i* of locus *G*, and genotype *j* of locus *H*, respectively. The penetrance is defined as a conditional probability of being affected on the genotype:

$f_{ij}=P(D=1\left|G=i,H=j)\right.$,

$f_{i\cdot}=P(D=1\left|G=i)\right.=\sum_{j} f_{ij}$,

$$f_{\cdot j}=P(D=1\left|H=j)\right.=\sum_{i} f_{ij}$$

Let$P_{ij}$, $P_{i\cdot}$ and $P_{\cdot j}$ denote *P*(*G*=*i*, *H*=*j*), *P*(*G*=*i*) and *P*(*H*=*j*) in the general population, respectively, and *K* denotes the population prevalence. We can show the genotype frequency distribution of loci *G* and *H* in cases and controls, respectively, in following tables:

| Cases | locus *H* | | | |
| --- | --- | --- | --- | --- |
| locus *G* | 0 (*bb*) | 1 (*Bb*) | 2 (*BB*) | margin |
| 0 (*aa*) | $P_{00}\frac{f_{00}}{K}$ | $P_{01}\frac{f_{01}}{K}$ | $P_{02}\frac{f_{02}}{K}$ | $P_{0\cdot}\frac{f_{0\cdot}}{K}$ |
| 1 (*Aa*) | $P_{10}\frac{f_{10}}{K}$ | $P_{11}\frac{f_{11}}{K}$ | $P_{12}\frac{f_{12}}{K}$ | $P_{1\cdot}\frac{f_{1\cdot}}{K}$ |
| 2 (*AA*) | $P_{20}\frac{f_{20}}{K}$ | $P_{21}\frac{f_{21}}{K}$ | $P_{22}\frac{f_{22}}{K}$ | $P_{2\cdot}\frac{f_{2\cdot}}{K}$ |
| margin | $P_{\cdot0}\frac{f_{\cdot0}}{K}$ | $P_{\cdot1}\frac{f_{\cdot1}}{K}$ | $P_{\cdot2}\frac{f_{\cdot2}}{K}$ | 1 |

| Controls | locus *H* | | | |
| --- | --- | --- | --- | --- |
| locus *G* | 0 (*bb*) | 1 (*Bb*) | 2 (*BB*) | margin |
| 0 (*aa*) | $P_{00}\frac{(1-f_{00})}{(1-K)}$ | $P_{01}\frac{(1-f_{01})}{(1-K)}$ | $P_{02}\frac{(1-f_{02})}{(1-K)}$ | $P_{0\cdot}\frac{(1-f_{0\cdot})}{(1-K)}$ |
| 1 (*Aa*) | $P_{10}\frac{(1-f_{10})}{(1-K)}$ | $P_{11}\frac{(1-f_{11})}{(1-K)}$ | $P_{12}\frac{(1-f_{12})}{(1-K)}$ | $P_{1\cdot}\frac{(1-f_{1\cdot})}{(1-K)}$ |
| 2 (*AA*) | $P_{20}\frac{(1-f_{20})}{(1-K)}$ | $P_{21}\frac{(1-f_{21})}{(1-K)}$ | $P_{22}\frac{(1-f_{22})}{(1-K)}$ | $P_{2\cdot}\frac{(1-f_{2\cdot})}{(1-K)}$ |
| margin | $P_{\cdot0}\frac{(1-f_{\cdot0})}{(1-K)}$ | $P_{\cdot1}\frac{(1-f_{\cdot1})}{(1-K)}$ | $P_{\cdot2}\frac{(1-f_{\cdot2})}{(1-K)}$ | 1 |

Hence, parameters in equation (1) could be expressed as

$P_{ij}^{A}=P_{ij}\frac{f_{ij}}{K}$, $P_{i\cdot}^{A}=P_{i\cdot}\frac{f_{i\cdot}}{K}$, $P_{\cdot j}^{A}=P_{\cdot j}\frac{f_{\cdot j}}{K}$

and

$P_{ij}^{N}=P_{ij}\frac{1-f_{ij}}{1-K}$, $P_{i\cdot}^{N}=P_{i\cdot}\frac{1-f_{i\cdot}}{1-K}$, $P_{\cdot j}^{N}=P_{\cdot j}\frac{1-f_{\cdot j}}{1-K}$

Hence,

$$\log\left(\frac{P_{ij}^{A}}{P_{i\cdot}^{A}P_{\cdot j}^{A}}\right)=\lambda_{ij}^{A}+\log\left(\frac{P_{ij}}{P_{i\cdot}P_{\cdot j}}\right)$$

$$\log\left(\frac{P_{ij}^{N}}{P_{i\cdot}^{N}P_{\cdot j}^{N}}\right)=\lambda_{ij}^{N}+\log\left(\frac{P_{ij}}{P_{i\cdot}P_{\cdot j}}\right)$$

$$\lambda_{ij}^{A}=\log\left(\frac{(f_{ij}/K)}{(f_{i\cdot}/K)(f_{\cdot j}/K)}\right)$$

$\lambda_{ij}^{N}=\log\left(\frac{(1-f_{ij})/(1-K)}{[(1-f_{i\cdot})/(1-K)]\cdot[(1-f_{\cdot j})/(1-K)]}\right)$.

Then, equation (1) can be expressed as

$$GenoCMI=\sum_{i} \sum_{j} P_{ij}\left[P_{A}\frac{f_{ij}\lambda_{ij}^{A}}{K}+(1-P_{A})\frac{(1-f_{ij})\lambda_{ij}^{N}}{1-K}+\left(P_{A}\frac{f_{ij}}{K}+(1-P_{A})\frac{(1-f_{ij})}{1-K}\right)\log\left(\frac{P_{ij}}{P_{i\cdot}P_{\cdot j}}\right)\right]$$

$$=\sum_{i} \sum_{j} P_{ij}\left[\left(\frac{(1-P_{A})(f_{ij}-1)}{(1-K)}\right)(\lambda_{ij}^{A}-\lambda_{ij}^{N})+\left(\frac{(f_{ij}-K)(P_{A}-K)}{K(1-K)}+1\right)\lambda_{ij}^{A}\right]$$

$+\sum_{i} \sum_{j} P_{ij}\left[\left(\frac{(f_{ij}-K)(P_{A}-K)}{K(1-K)}+1\right)\log\left(\frac{P_{ij}}{P_{i\cdot}P_{\cdot j}}\right)\right]$ (2)

In fact, if we define the relative risk (*RR*) of a genotype or genotype combination as the ratio between the penetrance of the given genotype and the population prevalence (also called the reference or baseline penetrance), $\lambda_{ij}^{A}$ could be referred as an information measure for the interaction between the two loci ^1^, which measures the log-scale of deviation between the risk of joint genotype against the additive risk of marginal genotypes (usually called main effects). Departure of $\lambda_{ij}^{A}$ from zero indicates the presence of interaction between loci *G* and *H*. Furthermore, we could obtain that,

$$\lambda_{ij}^{A}-\lambda_{ij}^{N}=\log\left(\frac{\frac{f_{ij}/(1-f_{ij})}{K/(1-K)}}{\frac{f_{i\cdot}/(1-f_{i\cdot})}{K/(1-K)}\cdot\frac{f_{\cdot j}/(1-f_{\cdot j})}{K/(1-K)}}\right)$$

$$=\log\left(\frac{OR_{ij}}{OR_{i\cdot}\cdot OR_{\cdot j}}\right)$$

where $\log\left(\frac{OR_{ij}}{OR_{i\cdot}\cdot OR_{\cdot j}}\right)$ can be shown as a measure of interaction between two loci *G* and *H* ^2, 3^. Hence, equation (2) could be reduced to

$$GenoCMI=\sum_{i} \sum_{j} P_{ij}\left[\left(\frac{(1-P_{A})(f_{ij}-1)}{(1-K)}\right)\log\left(\frac{OR_{ij}}{OR_{i\cdot}\cdot OR_{\cdot j}}\right)+\left(\frac{(f_{ij}-K)(P_{A}-K)}{K(1-K)}+1\right)\log\left(\frac{RR_{ij}}{RR_{i\cdot}\cdot RR_{\cdot j}}\right)\right]$$

$$+\sum_{i} \sum_{j} P_{ij}\left[\left(\frac{(f_{ij}-K)(P_{A}-K)}{K(1-K)}+1\right)\log\left(\frac{P_{ij}}{P_{i\cdot}P_{\cdot j}}\right)\right]$$

$$\approx P_{A}\sum_{i} \sum_{j} P_{ij}\left[RR_{ij}\log\left(\frac{RR_{ij}}{RR_{i\cdot}\cdot RR_{\cdot j}}\right)\right]$$

$+\sum_{i} \sum_{j} P_{ij}\left[\left(\frac{(f_{ij}-K)(P_{A}-K)}{K(1-K)}+1\right)\log\left(\frac{P_{ij}}{P_{i\cdot}P_{\cdot j}}\right)\right]$ (3)

(by using the approximation that *OR*≈*RR* if disease prevalence *K* is low, i.e., for a rare disease )

The second part of equation (3) is a function of *f_ij_* and $\log\left(\frac{P_{ij}}{P_{i\cdot}P_{\cdot j}}\right)$. The latter is a measure of dependence between loci *G* and *H* in general population, and hence could be viewed as a quantity for the population linkage disequilibrium (*LD*). In the case of two unlinked loci, this part is approximated to be zero, and

$GenoCMI\approx P_{A}\sum_{i} \sum_{j} P_{ij}\left[RR_{ij}\log\left(\frac{RR_{ij}}{RR_{i\cdot}\cdot RR_{\cdot j}}\right)\right]$ (4)

In a cohort study where *P_A_*=*K*, it can be easily seen that equation (3) becomes

$GenoCMI=K\sum_{i} \sum_{j} P_{ij}\left[RR_{ij}\log\left(\frac{RR_{ij}}{RR_{i\cdot}\cdot RR_{\cdot j}}\right)\right]+\sum_{i} \sum_{j} P_{ij}\log\left(\frac{P_{ij}}{P_{i\cdot}P_{\cdot j}}\right)$ (5)

**Conditional mutual information based on gametic disequilibrium (*GameteCMI*)**

Similarly, we consider two unlinked diallelic loci, *G* and *H*. Let alleles *A* and *B* be the risk alleles of loci *G* and *H*, respectively. Let *h_kl_* be a gamete of loci *G* and *H*, where *k* and *l* (*k*, *l* =0 and 1) indicates the carrier states of the risk alleles *A* and *B* in the gamete, respectively. The four possible gametes for the diallelic loci, *a-b*, *a-B*, *A-b* and *A-B*, are denoted by *h*_00_, *h*_01_, *h*_10_ and *h*_11_, respectively. Similar to equation (1), *GameteCMI* metric can be defined as:

$GameteCMI=\sum_{d} \sum_{k} \sum_{l} P(h_{kl},d)\log\left(\frac{P(h_{kl}\left|d\right.)}{P(G=k\left|d\right.)P(H=l\left|d\right.)}\right)$ (6)

where *P*(*G*=*k*|*d*) and *P*(*H*=*l*|*d*) are the frequencies of alleles *k* and *l* under disease status *d*, respectively. Similar to derivation of equations (2) and (3), the equation (6), approximately, can also be decomposed into two components,

$GameteCMI\approx P_{A}\sum_{k} \sum_{l} q_{kl}\left[RR_{kl}^{h}\log\left(\frac{RR_{kl}^{h}}{RR_{k\cdot}^{h}\cdot RR_{\cdot l}^{h}}\right)\right]$ $+\sum_{k} \sum_{l} q_{kl}\left[\left(\frac{(RR_{kl}^{h}-K)(P_{A}-K)}{(1-K)}+1\right)\log\left(\frac{q_{kl}}{q_{k\cdot}\cdot q_{\cdot l}}\right)\right]$ (7)

where $q_{kl}$, $q_{k\cdot}$ and $q_{\cdot l}$ are the frequencies of gamete *h_kl_*, and alleles *k* and *l* in general population, respectively. $RR_{kl}^{h}$, $RR_{k\cdot}^{h}$ and $RR_{\cdot l}^{h}$ are the relative risks of gamete *h_kl_*, and alleles *k* and *l*, respectively. Different from *GenoCMI*, the logarithm function in the second term of equation (7) explicitly describes the *LD* status between loci *G* and *H* in general population. For unlinked loci or loci without linkage disequilibrium, the second term equals to zero, because $D=q_{kl}-q_{k\cdot}\cdot q_{.l}=0$ in this case. Consequently, equation (7) can be reduced to

$GameteCMI\approx P_{A}\sum_{k} \sum_{l} q_{kl}\left[RR_{kl}^{h}\log\left(\frac{RR_{kl}^{h}}{RR_{k\cdot}^{h}\cdot RR_{\cdot l}^{h}}\right)\right]$ (8)

In a cohort study where *P_A_*=*K*, equation (7) becomes

$GameteCMI\approx K\sum_{k} \sum_{l} q_{kl}\left[RR_{kl}^{h}\log\left(\frac{RR_{kl}^{h}}{RR_{k\cdot}^{h}\cdot RR_{\cdot l}^{h}}\right)\right]+\sum_{k} \sum_{l} q_{kl}\log\left(\frac{q_{kl}}{q_{k\cdot}\cdot q_{\cdot l}}\right)$ (9)

Reference

**1.** Wu X, Jin L, Xiong M. Mutual information for testing gene-environment interaction. *PLoS One.* 2009;4(2):e4578.

**2.** Wu X, Dong H, Luo L, et al. A novel statistic for genome-wide interaction analysis. *PLoS Genet.* Sep 2010;6(9).

**3.** Ueki M, Cordell HJ. Improved statistics for genome-wide interaction analysis. *PLoS Genet.* Apr 2012;8(4):e1002625.
