# Supplementary material for: To Control False Positives in Gene-Gene Interaction Analysis: Two Novel Conditional Entropy-Based Approaches
Source: PLoS One. 2013 Dec 10;8(12):e81984. doi: 10.1371/journal.pone.0081984 (PMC3858311; doi:10.1371/journal.pone.0081984)
Supplement: Text S2 — Mathematical derivation of asymptotic distributions of GenoCMI and GameteCMI metrics. (DOC) [file pone.0081984.s010.doc]

**Text S2 Mathematical derivation of asymptotic distributions of *GenoCMI* and *GameteCMI* metrics**

**Null distribution of the *GenoCMI***

In the following derivations, we assume that two loci *G* and *H* are in linkage equilibrium, and both loci are in Hardy-Weinberg equilibrium. Their relative genotype frequency distribution in *N_A_* cases can be described as:

| Cases | locus *H* | | | |
| --- | --- | --- | --- | --- |
| locus *G* | 0 (*bb*) | 1 (*Bb*) | 2 (*BB*) | margin |
| 0 (*aa*) | $P_{00}^{A}$ | $P_{01}^{A}$ | $P_{02}^{A}$ | $P_{0\cdot}^{A}$ |
| 1 (*Aa*) | $P_{10}^{A}$ | $P_{11}^{A}$ | $P_{12}^{A}$ | $P_{1\cdot}^{A}$ |
| 2 (*AA*) | $P_{20}^{A}$ | $P_{21}^{A}$ | $P_{22}^{A}$ | $P_{2\cdot}^{A}$ |
| margin | $P_{\cdot0}^{A}$ | $P_{\cdot1}^{A}$ | $P_{\cdot2}^{A}$ | 1 |

The log-likelihood test for independence between columns (*H*) and rows (*G*), also called *G*-test, can be constructed:

$G_{case}=2\cdot\sum_{i} \sum_{j} O_{ij}^{A}\log\left(\frac{O_{ij}^{A}}{E_{ij}^{A}}\right)$ (1)

where $O_{ij}^{A}$ is the observed count for joint genotype *i* and *j*, and $E_{ij}^{A}$ is its expected frequency under the null hypothesis that the genotype distribution of loci *G* and *H* are independent. Note that the null hypothesis for *G*-test is in essence the same as that for mutual information, no loss of uncertainty of genotypes of locus *G* after introducing the information of locus *H*. If the null hypothesis for *G*-test holds, it is true for mutual information, too, or vice verse. Under this null hypothesis, $E_{ij}^{A}$ is the multiplication of the marginal counts of row *i* and column *j*, *i.e.*, $E_{ij}^{A}=N_{A}P_{i\cdot}^{A}P_{\cdot j}^{A}$.

In fact, the *G*-test for contingency table can be expressed in terms of mutual information. Let $O_{ij}^{A}=N_{A}P_{ij}^{A}$, the equation (1) becomes:

$$G_{case}=2\cdot N_{A}\cdot\sum_{i} \sum_{j} P_{ij}^{A}\log\left(\frac{P_{ij}^{A}}{P_{i\cdot}^{A}P_{\cdot j}^{A}}\right)$$

$$=2\cdot N_{A}\cdot MI_{case}$$

where $MI_{case}=\sum_{i} \sum_{j} P_{ij}^{A}\log\left(\frac{P_{ij}^{A}}{P_{i\cdot}^{A}P_{\cdot j}^{A}}\right)$ is the mutual information between loci *G* and *H* in cases. It is known that the null distribution for a log-likelihood ratio test (here, the *G*-test) is asymptotically a *χ*^2^ distribution. Hence, it can be easily seen that *MI_case_* asymptotically follows $\chi^{2}/2N_{A}$ distribution, with 4 degrees of freedom.

Similarly, we can show that the mutual information between genotypes of loci *G* and *H* in *N_N_* controls asymptotically follows:

$$MI_{control}=\sum_{i} \sum_{j} P_{ij}^{N}\log\left(\frac{P_{ij}^{N}}{P_{i\cdot}^{N}P_{\cdot j}^{N}}\right)\sim\chi_{4}^{2}/2N_{N}$$

Then, the conditional mutual information *GenoCMI* can be transformed as follows:

$$GenoCMI=\sum_{d} \sum_{i} \sum_{j} P(G=i,H=j,D=d)\log\left(\frac{P(G=i,H=j\left|D=d\right.)}{P(G=i\left|D=d\right.)P(H=j\left|D=d\right.)}\right)$$

$$=P_{A}\sum_{i} \sum_{j} P_{ij}^{A}\log\left(\frac{P_{ij}^{A}}{P_{i\cdot}^{A}P_{\cdot j}^{A}}\right)+(1-P_{A})\sum_{i} \sum_{j} P_{ij}^{N}\log\left(\frac{P_{ij}^{N}}{P_{i\cdot}^{N}P_{\cdot j}^{N}}\right)$$

$$=\frac{1}{2N_{T}}\left(2\cdot N_{A}\cdot MI_{case}+2\cdot N_{N}\cdot MI_{control}\right)$$

In a population-based study, cases and controls are unrelated. Hence, based on the additivity property of *χ*^2^ distributions, we obtain the null distribution of *GenoCMI*, which is asymptotically $\chi^{2}/2N_{T}$, where *N_T_* is the total sample size, and the degree of freedom is 8.

**Null distribution of the *GameteCMI***

Similarly to the above derivation, we construct the frequency distribution for gamete between loci *G* and *H* in cases.

| Cases | locus *H* | | |
| --- | --- | --- | --- |
| locus *G* | 0 (*b*) | 1 (*B*) | margin |
| 0 (*a*) | $h_{00}^{A}$ | $h_{01}^{A}$ | $h_{0\cdot}^{A}$ |
| 1 (*A*) | $h_{10}^{A}$ | $h_{11}^{A}$ | $h_{1\cdot}^{A}$ |
| margin | $h_{\cdot0}^{A}$ | $h_{\cdot1}^{A}$ | 1 |

The *G*-test for testing independence between alleles of loci *G* and *H* in *N_A_* cases can be defined as:

$$G_{case}=4\cdot N_{A}\cdot\sum_{i} \sum_{j} h_{ij}^{A}\log\left(\frac{h_{ij}^{A}}{h_{i\cdot}^{A}h_{\cdot j}^{A}}\right)\sim\chi_{1}^{2}$$

Intuitively, the coefficient here is 4 times *N_A_*, because one individual has two gametes. However, in practice, what we observe are genotypes instead of gamete. Although one individual has two gametes, for two loci with two alleles each, once one gamete is fixed, the other one can be completely determined. Therefore, the number of independent gamete for *N_A_* individuals is still *N_A_*, not 2*N_A_* (for detail, see ), which is also the reason that the original Wu et al statistics should be adjusted by half, as pointed out by Ueki et al. Hence, the MI based on gamete approximately follows $\chi_{1}^{2}/2N_{A}$, *i.e.*,

$$MI_{case}^{h}=\sum_{i} \sum_{j} h_{ij}^{A}\log\left(\frac{h_{ij}^{A}}{h_{i\cdot}^{A}h_{\cdot j}^{A}}\right)\sim\chi_{1}^{2}/2N_{A}$$

$$MI_{control}^{h}=\sum_{i} \sum_{j} h_{ij}^{N}\log\left(\frac{h_{ij}^{N}}{h_{i\cdot}^{N}h_{\cdot j}^{N}}\right)\sim\chi_{1}^{2}/2N_{N}$$

Finally, according to the additivity property of *χ*^2^ distributions, we derive that *GameteCMI* metric asymptotically follow $\chi^{2}/2N_{T}$, with 2 degrees of freedom, where the *N_T_* is total sample size, *i.e.*,

$$GameteCMI=\frac{1}{4N_{T}}\left(4\cdot N_{A}\cdot MI_{case}^{h}+4\cdot N_{N}\cdot MI_{control}^{h}\right)\sim\chi_{2}^{2}/2N_{T}$$

References

1. Rao S, Yuan M, Zuo X, Su W, Zhang F, et al. (2011) A novel evolution-based method for detecting gene-gene interactions. PLoS One 6: e26435.

2. Wu X, Dong H, Luo L, Zhu Y, Peng G, et al. (2010) A novel statistic for genome-wide interaction analysis. PLoS Genet 6.

3. Ueki M, Cordell HJ (2012) Improved statistics for genome-wide interaction analysis. PLoS Genet 8: e1002625.
